# Lattice-guided growth of dense arrays of aligned transition metal dichalcogenide nanoribbons with high catalytic reactivity

**DOI:** 10.1126/sciadv.adr8046

**Published:** 2025-01-08

**Authors:** Zongpeng Ma, Pablo Solís-Fernández, Kaito Hirata, Yung-Chang Lin, Keisuke Shinokita, Mina Maruyama, Kota Honda, Tatsuki Kato, Aika Uchida, Hiroto Ogura, Tomohiro Otsuka, Masahiro Hara, Kazunari Matsuda, Kazu Suenaga, Susumu Okada, Toshiaki Kato, Yasufumi Takahashi, Hiroki Ago

**Affiliations:** ^1^ Interdisciplinary Graduate School of Engineering Sciences, Kyushu University, Fukuoka 816-8580, Japan.; ^2^ Faculty of Engineering Sciences, Kyushu University, Fukuoka 816-8580, Japan.; ^3^ Department of Electronics, Graduate School of Engineering, Nagoya University, Nagoya 464-8603, Japan.; ^4^ Nanomaterials Research Institute, National Institute of Advanced Industrial Science and Technology (AIST), Tsukuba 305-8565, Japan.; ^5^ The Institute of Scientific and Industrial Research (ISIR-SANKEN), Osaka University, Osaka 567-0047, Japan.; ^6^ Institute of Advanced Energy, Kyoto University, Kyoto 611-0011, Japan.; ^7^ Department of Physics, Graduate School of Pure and Applied Sciences, University of Tsukuba, Tsukuba 305-8571, Japan.; ^8^ Graduate School of Engineering, Tohoku University, Sendai 980-8579, Japan.; ^9^ Research Institute of Electrical Communication, Tohoku University, Sendai 980-8577, Japan.; ^10^ Advanced Institute for Materials Research (AIMR), Tohoku University, Sendai 980-8577, Japan.; ^11^ Center for Science and Innovation in Spintronics, Tohoku University, Sendai 980-8577, Japan.; ^12^ Center for Emergent Matter Science, RIKEN, Saitama 351-0198, Japan.; ^13^ Faculty of Advanced Science and Technology, Kumamoto University, Kumamoto 860-8555, Japan.; ^14^ Institute of Industrial Nanomaterials, Kumamoto University, Kumamoto 860-8555, Japan.; ^15^ WPI Nano Life Science Institute (WPI-NanoLSI), Kanazawa University, Kanazawa 920-1192, Japan.; ^16^ Center for Semiconductor and Device Ecosystem (CSeDE), Kyushu University, Fukuoka 816-8580, Japan.

## Abstract

Transition metal dichalcogenides (TMDs) exhibit unique properties and potential applications when reduced to one-dimensional (1D) nanoribbons (NRs), owing to quantum confinement and high edge densities. However, effective growth methods for self-aligned TMD NRs are still lacking. We demonstrate a versatile approach for lattice-guided growth of dense, aligned MoS_2_ NR arrays via chemical vapor deposition (CVD) on anisotropic sapphire substrates, without tailored surface steps. This method enables the synthesis of NRs with widths below 10 nanometers and longitudinal axis parallel to the zigzag direction, being also extensible to the growth of WS_2_ NRs and MoS_2_-WS_2_ heteronanoribbons. Growth is influenced by both substrate and CVD temperature, indicating the role of anisotropic precursor diffusion and substrate interaction. The 1D nature of the NRs was asserted by the observation of Coulomb blockade at low temperatures. Pronounced catalytic activity was observed at the edges of the NRs, indicating their promise for efficient catalysis.

## Teaser

Dense arrays of ultrathin TMD nanoribbons show promise for next-generation catalysis and nanoelectronics.

## INTRODUCTION

Transition metal dichalcogenides (TMDs), a family of two-dimensional (2D) semiconducting materials, have been gaining interest for their potential in post-Si electronic devices to extend Moore’s law (*1*, *2*). In particular, TMDs are expected to play an important role in future logic circuits, such as gate-all-around and nanosheet transistors, offering ultrathin 2D channels with high carrier mobility, in contrast to conventional Si electronics (*3*). Such applications require the use of narrow TMD channels with atom-level thickness, with nanoribbons (NRs), 1D forms of 2D materials, fulfilling this requirement. Moreover, reducing the dimensionality from 2D to 1D substantially alters the electronic structure of the materials, leading to unique physical properties, such as edge- and width-dependent band structure (*4*), metallic edge states (*5*), magnetic ordering (*5*, *6*), piezoelectricity (*7*), and robust carrier mobility (*8*). Furthermore, the high edge-to-area ratio of NRs can enhance their catalytic activity (*9*, *10*).

TMD NRs are obtained via two main approaches, namely, top-down and bottom-up methods. Top-down approaches typically rely on lithographic techniques (*11*, *12*), allowing position-selective patterning but suffering from low throughputs and difficulties in controlling the edge structures, and with ribbon widths limited by the resolution of lithography and etching techniques. Some studies have used these methods to create TMD NR–based field-effect transistors (FETs), providing initial insights into their electronic properties (*13*, *14*) and proving their 1D nature (*15*). Chemistry-based top-down methods offer higher throughput but introduce a large amount of defects (*16*), whereas mechanical exfoliation from bulk materials can produce aligned NR arrays but lacking control over width and thickness (*17*). Chemical vapor deposition (CVD) is a promising bottom-up approach for large-scale synthesis of TMD NRs with controlled width and orientations. However, CVD typically yields triangular 2D grains with zigzag (ZZ) edges (*4*, *18*), with only limited studies on the synthesis of TMD NRs by CVD (*19*–*23*). Vapor-solid-liquid CVD growth of MoS_2_ NRs has been demonstrated using metal alloy and Ni nanoparticles (*19*, *20*). In addition, MoS_2_ NRs have been produced by bottom-up synthesis using step-terrace structures specifically formed on surfaces like Au (*23*, *24*) and β-Ga_2_O_3_ (*22*) that serve as nucleation points and assist the growth to form ribbon-like structures. However, this method relies on very specific substrates with special miscut angles to obtain the tailored steps. Furthermore, precise control over the structure of the NR edges is still challenging in this step-templated growth mode, often leading to sawtooth NR edges (*22*). Hence, there is a pressing need for a more versatile approach to produce narrow, high-density aligned TMD NRs.

Here, we demonstrate a self-aligned growth mode of TMD NRs by CVD, leveraging lattice guidance on low-symmetry sapphire (α-Al_2_O_3_) substrates. Such kind of substrates have been previously used to grow aligned carbon nanotubes (*25*, *26*) and graphene NRs (*27*) and enabled growing aligned rectangular grains of MoS_2_ and WS_2_ (*28*). The anisotropic surface atomic arrangements of a- and r-plane sapphire substrates control the growth directions of MoS_2_, yielding narrow and highly aligned NR arrays. This alignment is particularly interesting as it paves the way for potential industrial scaling of the growth process, a crucial step toward practical applications of these NRs. Temperature and a proper exposure to the precursors, in addition to the substrate, were found to be key parameters for 1D growth, with density functional theory (DFT) calculations hinting to the role of anisotropic precursor diffusion. The NRs display remarkable carrier mobility, even at narrower widths, underscoring their exceptional quality (*8*), with the observation of Coulomb blockade at low temperatures confirming the 1D nature of the NRs (*15*). The method can be also developed to the synthesis of MoS_2_-WS_2_ hetero-NRs (hNRs). Furthermore, we revealed that edges of MoS_2_ NRs act as highly active catalyst sites for hydrogen evolution reaction (HER). Our findings offer an original pathway to fabricate large-scale, aligned TMD NR arrays for catalysis and electronics.

## RESULTS

### Self-aligned growth of TMD NRs

**Fig. 1.**
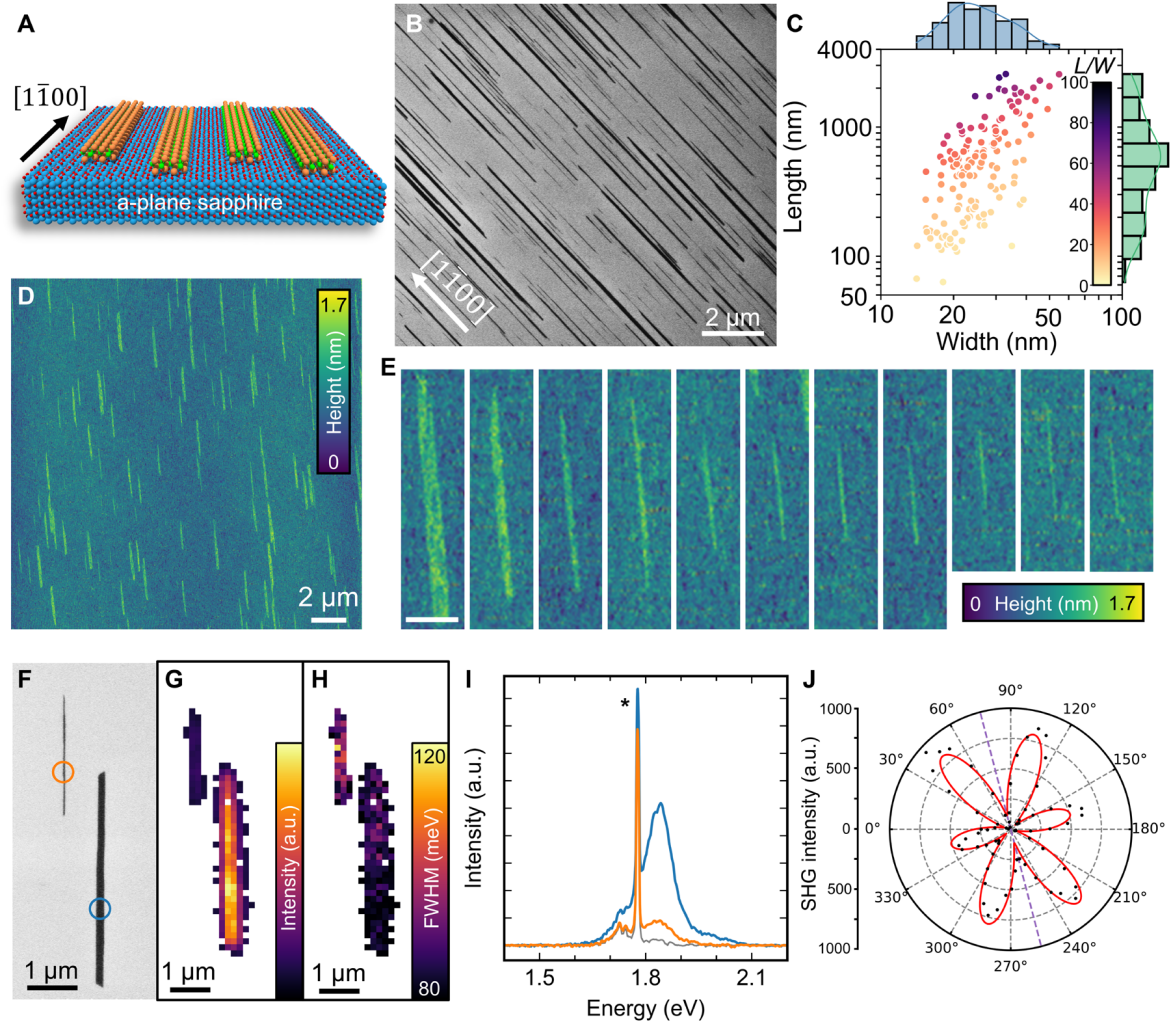
Lattice-guided growth of MoS_2_ NRs. (**A**) Model of the aligned MoS_2_ NRs on a-sapphire. (**B**) SEM image of the as-grown NRs. (**C**) Distribution of the width versus the length of ~150 NRs, measured by SEM. The color of the markers indicates the aspect ratio (*L*/*W*) of the given NR. (**D**) AFM image of the as-grown NRs. (**E**) High-magnification AFM images of isolated narrow NRs, with widths down to ~24 nm. Scale bar, 500 nm. SEM image (**F**) and corresponding PL mapping of the A-exciton intensity (**G**) and FWHM (full width at half maximum) (**H**) of two NRs of widths 40 nm (left) and 130 nm (right). a.u., arbitrary units. (**I**) PL spectra of the narrow (orange) and wide (blue) ribbons, taken at the positions marked in (F). The spectrum of bare sapphire (gray) is shown for comparison, and the signal from the sapphire is marked with an asterisk. (**J**) Polar plot of the polarization-resolved SHG signal intensity of the MoS_2_ NRs. The black dots correspond to the experimental data, whereas the red line is the fit. The longitudinal axis of the NR is indicated by the purple dotted line. The PL in (G) to (I) was collected at room temperature using an excitation wavelength of 532 nm.

The symmetry of crystals can be used to grow low-dimensional materials with controlled shapes and alignments (*27*, *29*–*31*). Here, we introduce a substrate lattice-guided method to produce aligned TMD NRs on a-plane sapphire $\left(11\bar{2}0\right)$, which has a twofold symmetry. The growth is tuned by the temperature and controlled exposure to precursors (sulfur and MoO_3_ or WO_3_ to grow MoS_2_ or WS_2_, respectively) during the CVD (Fig. 1A). Further experimental details are described in the Materials and Methods section. Figure 1B and fig. S1 show scanning electron microscopy (SEM) images of regions with dense arrays of aligned MoS_2_ NRs, grown at 1100°C on a-plane sapphire. The selection of the substrate is critical to produce NRs, with the same growth conditions producing triangular flakes on c-plane sapphire (fig. S2). As will be shown later, the NRs are oriented along the $\left[1\bar{1}00\right]$ direction of the sapphire substrate. Here, the choice of substrate, growth temperature, and amount of precursor are all indispensable for the successful growth of NRs. These ribbons can have lengths up to a few tens of micrometers and widths that are below 10 nm. The distribution of the width and length of ~150 NRs is shown in Fig. 1C. These data, collected from the SEM images in fig. S3, evidence a positive correlation between the length (*L*) and width (*W*) of the NRs, with aspect ratios (*L*/*W*) of up to ~80. This choice of SEM imaging here represents a balance between the greater accuracy of other microscopic techniques and the high throughput of SEM, although it prevents the inclusion of both the narrowest and longest NRs. To delve deeper into the characteristics of the narrowest NRs, atomic force microscopy (AFM) images of the NRs were taken, as displayed in Fig. 1 (D and E). The width of these NRs and the AFM image of the whole area are shown in fig. S4. The NRs exhibited apparent widths down to ~24 nm by AFM. However, as will be discussed afterward, scanning transmission electron microscopy (STEM) images revealed the existence of NRs narrower than 10 nm. This suggests that the widths measured by AFM are likely overestimated due to the effect of tip convolution.

The single-layer nature of the NRs was confirmed by AFM (fig. S4, B and C), with a measured average thickness of ~4.5 Å, and further supported by optical measurements. The observation of the Raman shift of the characteristic A_1g_ and E_2g_ peaks of MoS_2_, along with their relative distance (~20 cm^−1^), indicates that the NRs are single layered (fig. S5) (*32*). Photoluminescence (PL) mappings were collected from a representative area populated with NRs (Fig. 1, F to I), as well as from a distinct area that contained both triangular MoS_2_ flakes and NRs (fig. S6). The mappings reveal a lower PL intensity for the NRs compared with the triangular flakes. This weaker PL of the NR can be attributed to the larger laser spot size (~1 μm) relative to the NR width, rather than the NR being multilayer. This is also supported by the observation that narrower NRs tend to exhibit lower PL intensities (Fig. 1, G and I). The PL energy is also shifted to higher energies for the NRs, which can be attributed to a higher strain from the substrate coupled to the quantum confinement effect arising from the reduced dimensionality of the NRs (*33*). The narrower PL of the NRs (fig. S6D) suggests higher crystalline quality compared to the triangular flakes (*34*).

The orientation of the NRs was determined using polarization-resolved second-harmonic generation (SHG), a technique that can probe the structural symmetry of TMDs (*17*, *35*). The parallel polarization component of the SHG signal reaches a maximum intensity when the polarization of the incident laser aligns with the armchair direction of the MoS_2_ (*35*). Therefore, the SHG signal of the NRs, depicted in Fig. 1J, indicates that the longitudinal axis of the NRs is parallel to the MoS_2_ ZZ direction. Instead of the sixfold symmetric signal expected from MoS_2_ flakes, we observed a decrease in the intensity of the lobes in the direction perpendicular to the longitudinal axis of the NRs. This anomaly indicates a deviation from the usual single cos^2^ component used to fit the SHG signal of MoS_2_ flakes (*35*). A more accurate fit for the NRs is achieved with a product of two cos^2^ components. One of these components accounts for the sixfold symmetry of the MoS_2_ lattice and another for the twofold symmetry (fig. S7). Small asymmetries of the lobes in exfoliated TMD NRs have recently been attributed to strain along the perpendicular direction of the NR axis (*17*). In addition, these asymmetries can also be ascribed to the 1D nature of the NRs (*36*).

### Analysis of edges and orientation of the NRs

**Fig. 2.**
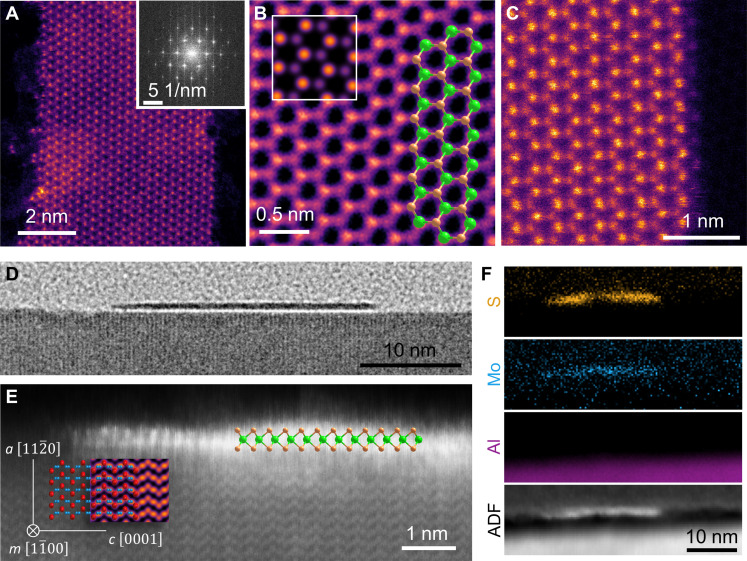
Structure and alignment of the MoS_2_ NRs. (**A** to **C**) Top-view STEM images of a 5.7-nm-wide MoS_2_ NR supported on graphene. (A) Low-magnification image of the NR. The inset corresponds to the FFT. (B) FFT-filtered atomic-resolution STEM image of the NR. The top-left inset shows a simulated STEM image of MoS_2_. A model of the MoS_2_ lattice is overlapped on the right side, with Mo and S atoms colored in green and orange, respectively. (C) High-resolution image of the Mo–S terminated ZZ edge of the NR. The row of single S atoms at the edge is clearly visible. (**D**) Cross-sectional TEM image of a 26-nm-wide NR on the sapphire. (**E**) STEM image of the S-terminated edge of the NR in (D). A schematic of the MoS_2_ lattice (Mo, green; S, orange) is superimposed on the right side of the NR in (E). A schematic of the cross section of the sapphire (m-plane, with Al and O atoms in blue and red, respectively) and a simulated STEM image are superimposed at the bottom-left side in (E). (**F**) EELS element mappings of S (158.0 to 203.5 eV), Mo (248.8 to 276.3 eV), and Al (68.8 to 128.3 eV) and the corresponding annular dark-field (ADF) image.

The atomic structure of the NR and their edges and their orientation with respect to the sapphire substrate were characterized by STEM. Figure 2A displays a top-view STEM image of an ~5.7-nm-wide NR supported on single-layer graphene (SLG), with the inset showing the fast Fourier transform (FFT) of the image. The STEM analysis confirms that the NR consists of a single layer with a 1H structure and indicates that the longitudinal axis of the NRs is aligned parallel to the ZZ direction of the MoS_2_ lattice (*4*, *23*). These observations are consistent with the AFM and SHG results discussed previously. A high-resolution image of the center of the NR is presented in Fig. 2B, in which the absence of defects reflects the high crystalline quality of the NRs.

An examination of the edges of the NRs reveals differences between the two sides of the ribbon. As seen in the model overlapped in Fig. 2B, the right and left edges of the ribbon are expected to be terminated by Mo (ZZ-Mo) and S_2_ (ZZ-S_2_) atoms, respectively. Figure 2C shows that the ZZ-Mo edge is relatively straight, and a closer inspection shows that the Mo atoms at the edge are terminated by single S atoms [ZZ-Mo (S)] (see fig. S8, A to C). The S termination of the ZZ-Mo edge has been previously reported for the edges of MoS_2_ nanoclusters (*37*) and suggests a sulfur-rich environment during the growth of the NRs (*38*). In contrast, analysis of the edge on the ZZ-S_2_ side of the NR reveals a reconstruction of the edge structure (fig. S8D). The ZZ-S_2_ edge maintains a global direction that is parallel to the ZZ direction of MoS_2_, although it is locally terminated with a mixture of both ZZ-S_2_ and ZZ-Mo (S) sections forming angles of 60° between them. This is in accordance with calculations showing that ZZ-Mo (S) edges are expected to be more stable than ZZ-S_2_ edges in S-rich growth environments (*39*, *40*). A similar behavior has been reported for the case of MoSe_2_ NRs, for which the Mo edge is smoother than the Se edge (*41*). This reconstruction can be attributed to the higher stability of the ZZ-Mo (S) sections compared to the ZZ-S_2_ under the actual growth conditions in a sulfur-rich environment (*38*).

The alignment direction of the NRs with respect to the sapphire substrate was determined from cross-sectional high-resolution images of a 26-nm-wide NR sectioned perpendicularly to its longitudinal axis. A wide transmission electron microscopy (TEM) view of the NR can be seen in Fig. 2D, with a detailed STEM image of the ZZ-Mo (S) edge and the substrate shown in Fig. 2E. The elemental composition of the NR measured by electron energy-loss spectroscopy (EELS) mappings indicates the presence of MoS_2_ on the top of the sapphire surface (Fig. 2F). In addition, EELS spectra of the MoS_2_ (fig. S9) show the characteristic S and Mo edges. The sapphire crystal face shown in the cross-sectional images is representative of the m-plane (*18*), proving that the NRs grow parallel to the $\left[1\bar{1}00\right]$ direction. This observation confirms the lattice-oriented growth of the NRs, which can be attributed to the linear atomic arrangement of the O atoms in the surface of the a-plane for each of the two O layers (fig. S10). The factors enabling the linear growth of the NRs are discussed in the next section.

### Role of growth temperature and substrate

**Fig. 3.**
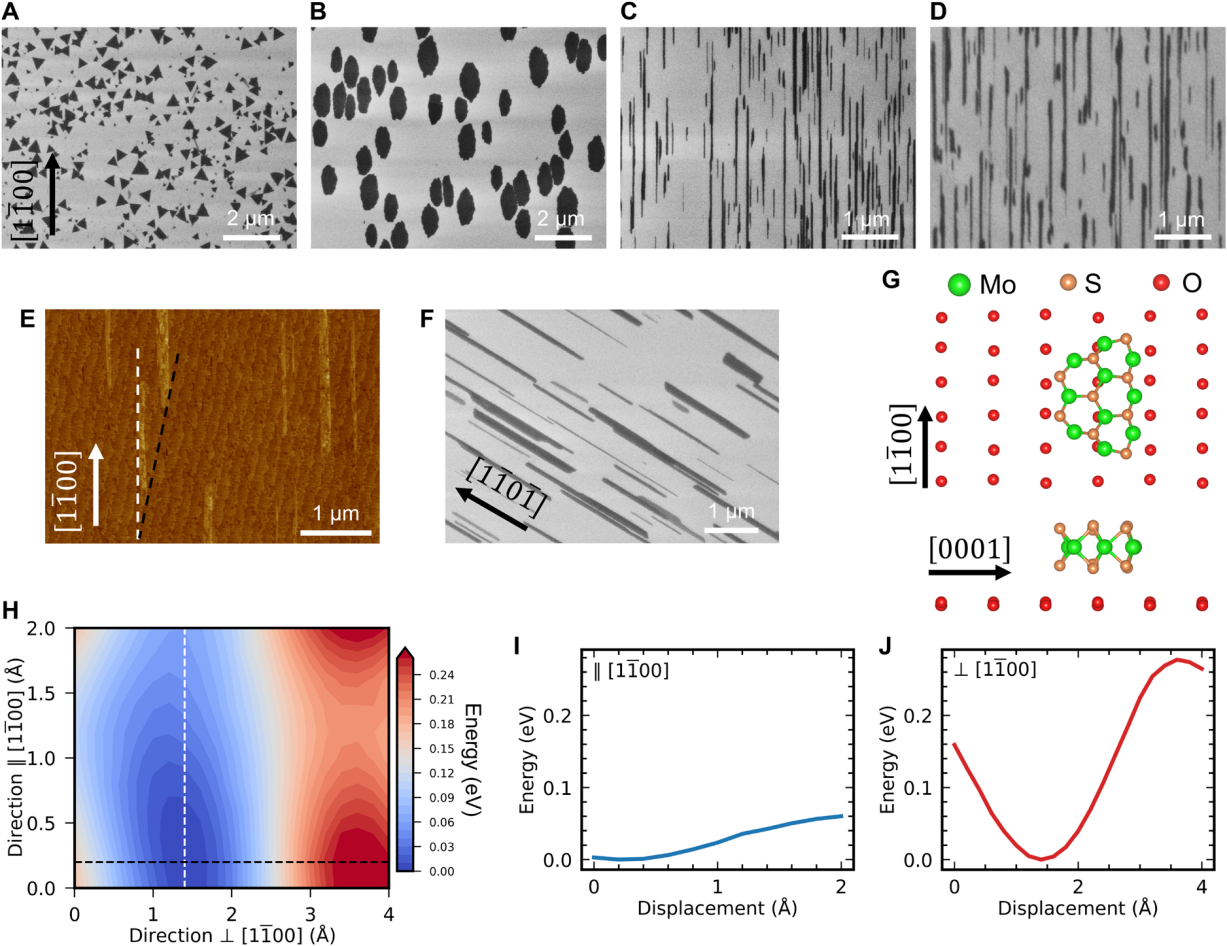
Growth mechanism of aligned MoS_2_ NRs. (**A** to **D**) Temperature dependence of the morphological structure of MoS_2_ grown at 750°C (A), 950°C (B), 1100°C (C), and 1200°C (D). The increase in the CVD temperature produces both an alignment and a 1D constrained growth. (**E**) AFM image showing a relative alignment of the MoS_2_ NRs (white dashed line) and the atomic steps of the sapphire surface (black dashed line) of around 13°. (**F**) MoS_2_ NRs grown on r-plane sapphire. (**G**) Atomic model used for the DFT calculations viewed from the top $\left[11\bar{2}0\right]$ direction (upper schematic) and the side $\left[1\bar{1}00\right]$ direction (lower schematic) of the a-plane sapphire lattice. The NR-like MoS_2_ flake is oriented with the ZZ direction parallel to $\left[1\bar{1}00\right]$. For simplicity, only the top O atoms of the sapphire are displayed. (**H**) Contour plot of the total energy of the system shown in (G) for different positions of the NR-like MoS_2_ flake over the sapphire. (**I** and **J**) Energy profiles taken along the white (I) and black (J) dashed lines in (H), crossing at the most stable configuration.

We found that the growth temperature is a critical factor in producing NRs, observing a transition from the growth of randomly oriented MoS_2_ flakes to aligned NRs as the temperature increased. As depicted in Fig. 3A, submicrometer-sized, randomly oriented MoS_2_ flakes are obtained at lower temperatures (750°C). As will be shown below, the small size of the flakes is related with a controlled exposure to the precursors, which plays an essential role in the growth of NRs. When the temperature is raised to 950°C, the flakes begin to show an alignment along the $\left[1\bar{1}00\right]$ direction of sapphire, and their shape transitions from the triangles to irregular grains (Fig. 3B). At 1100°C, sharp and narrow NRs are produced (Fig. 3C). This temperature dependence highlights the importance of the anisotropic diffusion of intermediate species on the a-plane surface. At high temperatures, the surface diffusion is enhanced, resulting in the formation of MoS_2_ NRs. However, further increasing the temperature results in an increased amount of precursors on the sapphire surface. Hence, increasing the temperature up to 1200°C did not produce notably narrower NRs (Fig. 3D). Instead, it increased the amount of MoO*_x_* species on the sapphire substrate, producing thicker NRs, a higher density of NRs, and the formation of thick Mo-based by-products in several areas of the substrate (fig. S11). At these high temperatures, the growth of some NRs along directions different to $\left[1\bar{1}00\right]$ was also evident.

Previous research highlighted the role of temperature in transitioning from the growth of triangular to rectangular-shaped MoS_2_ flakes on a-plane sapphire by face-down exposure to the precursors (*28*). However, the high temperatures (~1100°C) necessary to grow NRs led to enhanced sublimation of MoO_3_, resulting in an uncontrolled growth and the formation of thick Mo-based materials (fig. S12A). To decrease the width of the MoS_2_ flakes and successfully produce NRs, it was essential to increase the CVD temperature while limiting the amount of MoO*_x_* species on the sapphire substrate. This was achieved by positioning the sapphire downstream and far from the precursors (fig. S12B).

Despite the presence of steps on the a-plane sapphire surface, attributed to minor miscut angles, their influence on the growth and alignment of the MoS_2_ NRs was found to be negligible. This is evident from the AFM images, which show a misalignment of ~13° between the NRs and the steps (Fig. 3E). This observation confirms that the 1D growth is governed by the lattice structure of the sapphire, rather than the surface steps. To confirm the role of the low symmetry of the substrate, the growth of MoS_2_ NRs was also performed on r-plane sapphire $\left(1\bar{1}02\right)$ (*25*, *27*). The MoS_2_ NRs grew along the preferential direction $\left[1\bar{1}0\bar{1}\right]$ of the sapphire, rather than following the direction of the sapphire steps (Fig. 3F and fig. S13). In contrast, the growth of TMD NRs on other substrates, such as faceted Au, is reported to be initiated and guided by steps on the surface (*23*). The lattice-guided growth ensures a more uniform and predictable alignment of the NRs, which is critical for the reproducibility and scalability of the synthesis. In addition, lattice-guided growth is less dependent on the surface morphology of the substrate, potentially allowing for a wider range of substrate choices and simplifying the preparation process.

DFT calculations were performed to understand the relationship between the aligned 1D growth and the crystal lattice of the substrate. A small, NR-like MoS_2_ flake with a trapezoidal shape and ZZ edges was positioned atop an O-terminated a-plane sapphire lattice. A simplified model of the structure containing only the top layer of O atoms of the sapphire is shown in Fig. 3G, with examples of the whole models shown in fig. S14. The total energy was computed across a range of relative positions of the flake over the sapphire lattice (Fig. 3H and movie S1). The calculations revealed that the relative position between the Mo atoms in the MoS_2_ flake and the surface atoms of the sapphire is important for the stability of the system. Minimum energies were achieved when the Mo atoms were in close proximity to the O atoms of the sapphire, in which a substantial hybridization between Mo 4d and O 2p orbitals occurred. This strong interaction between the Mo atoms and the O atoms stabilizes the system and lowers the total energy.

Furthermore, the calculations indicated that the barrier energies for the diffusion of the flakes are smaller in the $\left[1\bar{1}00\right]$ direction of the sapphire. On the other hand, the barrier in the [0001] direction (perpendicular to $\left[1\bar{1}00\right]$) is more than five times larger (see Fig. 3, I and J). These directions correspond to those in which the number of Mo–O pairs remain almost constant ($\left[1\bar{1}00\right]$) and to that where there is a substantial modulation in the atomic arrangement between the two lattices ([0001]). A preferential diffusion of S_2_ monomers along the $\left[1\bar{1}00\right]$ direction of the a-plane has also been recently reported (*31*), leading to the preferential growth of WS_2_ trapezoidal flakes along such a direction, whereas similar anisotropic diffusion barriers have been found for the aligned growth of WS_2_ on m-plane quartz (*42*). This suggests that precursor diffusion is more favorable in the $\left[1\bar{1}00\right]$ direction, resulting in preferred growth along this axis.

### Growth of hNRs

The versatility of our method allows for the growth of other kinds of NRs, such as WS_2_ NRs, as shown in fig. S15, through a simple change of the precursor. By adjusting the composition of the precursor during the growth or at a subsequent growth step, the lattice-guided CVD allows to grow hNRs composed of lateral heterostructures. These hNRs are of considerable interest due to their potential in advanced electronic and optoelectronic devices (*43*). The seamless stitching within a single atomic layer in these lateral heterostructures offers opportunities for engineering of the band structure and their properties, making them promising candidates for applications in nanoelectronics and optoelectronic applications (*2*, *44*, *45*). The ability to grow such structures in an aligned manner further enhances their potential for scalable device integration.

**Fig. 4.**
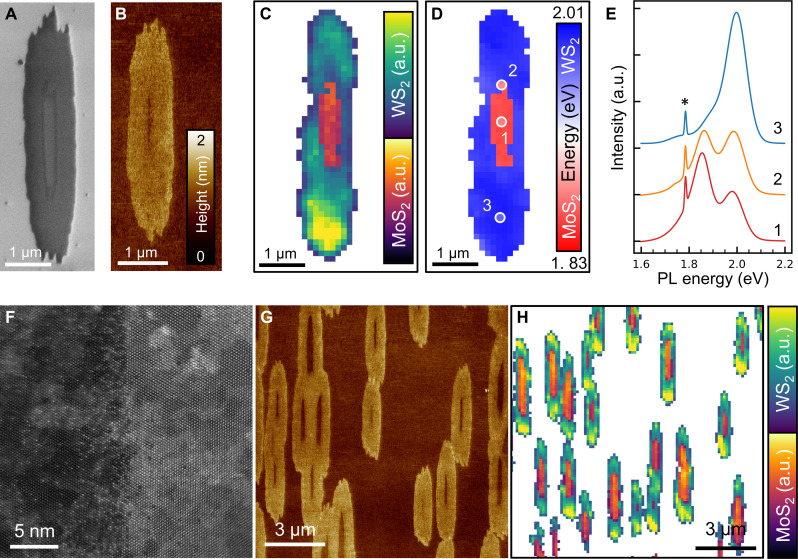
MoS_2_-WS_2_ hNRs. (**A** and **B**) SEM (A) and AFM (B) images of isolated MoS_2_-WS_2_ lateral hNRs. (**C** and **D**) Mappings of the PL intensity (C) and the PL energy (D) of a MoS_2_-WS_2_ hNR. (**E**) PL spectra of the hNR collected at the points marked in (D). The asterisk in (E) indicates the signal from the sapphire substrate. (**F**) STEM image of the MoS_2_-WS_2_ interface, with the MoS_2_ and the WS_2_ on the left and right sides, respectively. (**G** and **H**) Wide-area AFM image (G) and PL intensity mapping (H) of hNRs. Note that the SEM, AFM, and PL areas for both the isolated hNRs and the wide-area regions correspond to representative areas but are not identical. The PL data were collected at room temperature using an excitation wavelength of 532 nm.

Figure 4 (A and B) depicts SEM and AFM images of isolated in-plane MoS_2_-WS_2_ hNRs, respectively, which were obtained by switching the precursor from MoO_3_ to WO_3_. In both images, two distinct regions can be discerned: one at the center of the hNR and the other surrounding it. The PL mapping collected in one of such hNRs clearly shows that the center is composed of MoS_2_, with the surrounding material being WS_2_ that laterally extended from the original MoS_2_ seed during the second stage (Fig. 4, C and D). The presence of these distinct peaks, without any intermediate peaks characteristic of Mo*_x_*W_(1−*x*)_S_2_ alloys, proves the formation of a lateral heterojunction similar to those observed for large flakes (*44*–*46*). Individual PL spectra collected at the distinct regions of the hNR (MoS_2_, WS_2_, and the interface) are shown in Fig. 4E, whereas the evolution of the spectra along two different directions is shown in fig. S16. Given that the components of the hNR are smaller than the size of the PL laser spot (~1 μm), our setup does not allow for a strict differentiation of pure MoS_2_ and WS_2_ areas within a single spectrum. Only pure WS_2_ can be discerned at the ends of the ribbon for hNRs longer than a few micrometers (top and bottom areas of the hNR in fig. S16D). The Raman mapping of a broader hNR, shown in fig. S17, allows us to observe a sharp transition between the regions with WS_2_ peaks and with MoS_2_ peaks. This, along with the distinct PL emissions observed in fig. S16, proves the formation of well-defined MoS_2_ and WS_2_ domains within the hNRs, rather than the formation of alloyed regions (*46*). To further characterize the MoS_2_-WS_2_ interface at the atomic scale, we performed high-resolution STEM imaging (Fig. 4F and fig. S18). The results revealed a narrow diffusion region of ~6 nm at the interface, in which primarily W atoms (brighter in the STEM images) diffused into the MoS_2_ lattice, forming substitutional defects. This limited interdiffusion is consistent with our growth process, in which MoS_2_ is grown first. The diffusion region tends to be narrower at the smoother ZZ-Mo (S) edge of the MoS_2_ NRs, compared to the ZZ-S_2_ edge (fig. S18, A and B). The edge-dependent behavior suggests that refining growth conditions for both the MoS_2_ and WS_2_ components could potentially minimize interdiffusion, leading to even sharper interfaces. For instance, this could be achieved by producing straighter edges of the initial MoS_2_ NR or by decreasing the processing temperature for the subsequent growth of WS_2_. Despite this localized mixing at the atomic scale, the overall structure maintains distinct MoS_2_ and WS_2_ domains, as evidenced by PL and Raman, and by EELS (fig. S18C) of the different regions. The STEM analysis also revealed that the WS_2_ domain is single crystalline and maintains the same crystallographic orientation as the MoS_2_ domain, indicating a seamless stitching between the two materials.

SEM imaging and PL mappings of wide areas (Fig. 4, G and H, and fig. S19) demonstrate that the growth of the MoS_2_-WS_2_ hNRs is uniform across the sapphire substrate, with all the original MoS_2_ NRs acting as seeds to form the in-plane hNRs. The proposed method can be used to produce A-B-A (with A, B: MoS_2_, WS_2_, MoSe_2_ …) heterojunctions along the longitudinal axis of the hNR and with nanometer-sized widths determined by the initial width of the NR acting as a seed (*45*, *47*).

In summary, the growth of hNRs proves that our method allows for precise control over both dimensionality and composition, resulting in truly 1D TMD heterostructures. This opens up the possibility to design complex nanostructures with tailored properties.

### Edge-dominated HER catalytic activity

TMDs are known as promising catalysts for HER, with reactivity being higher at the edges compared to the basal planes (*48*). Because of their high edge-to-area ratio, NRs are expected to be ideal candidates for high HER activity compared to 2D flakes. To investigate the local HER activity of MoS_2_ NRs, we used high-resolution scanning electrochemical cell microscopy (SECCM) (*48*). This technique enables the spatial investigation of the electrochemical activity, distinguishing the activity between the edges and central regions of the NRs and offering insights into the spatial distribution of catalytic performance.

**Fig. 5.**
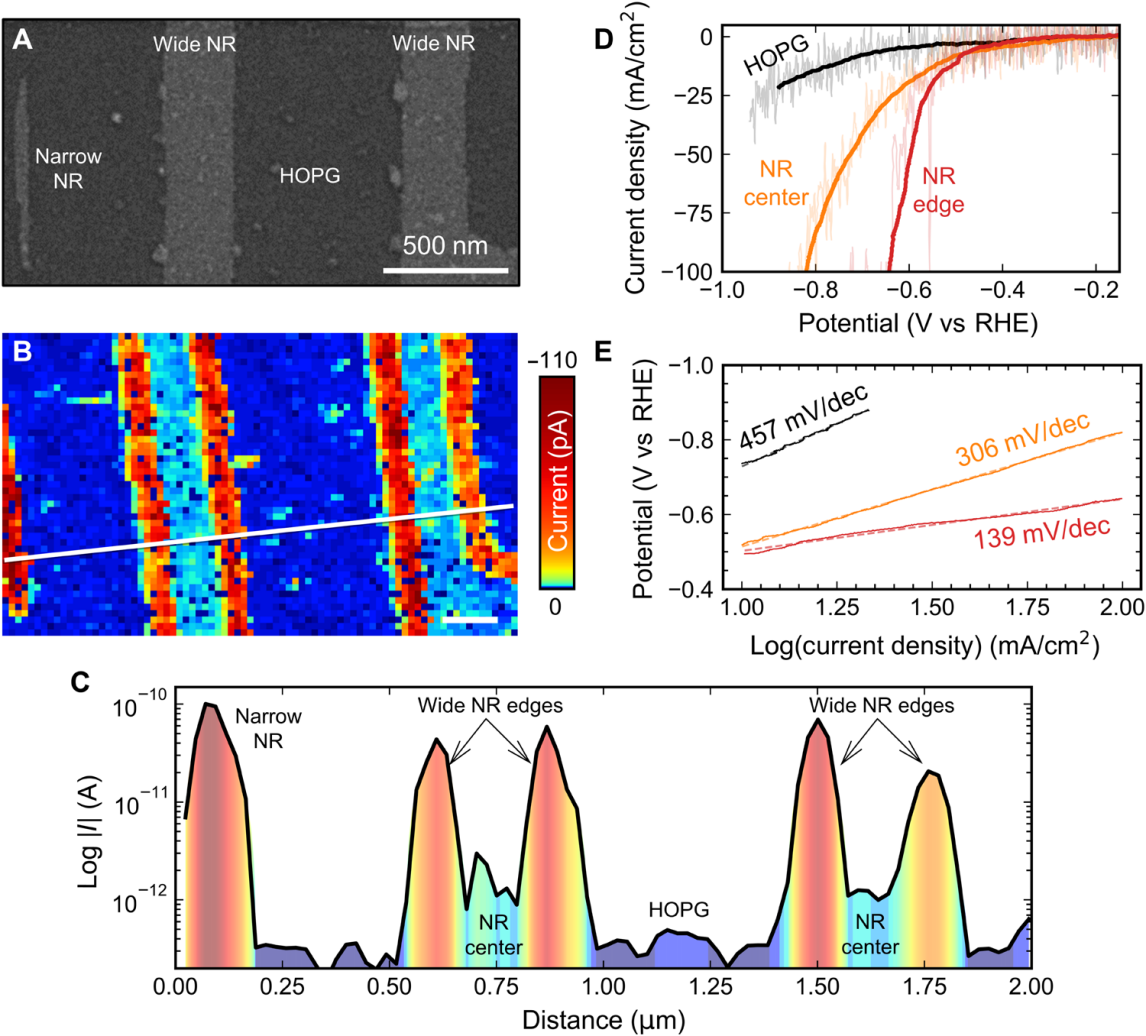
Catalytic activity of the MoS_2_ NRs. (**A** and **B**) SEM image (A) and the corresponding HER current (−0.95 V versus RHE) mapping (B) of a narrow NR and two wide NRs supported on HOPG. Scale bar in (B), 200 nm. (**C**) Electrochemical current along the white line indicated in (B). The labels indicate the nature of the corresponding regions. The shown profile is averaged from five parallel profiles to decrease the noise. (**D** and **E**) Overpotential (D) and Tafel slope (E) for HOPG (black) and for the center (orange) and edge (red) of a wide NR. The overpotential graph (D) displays both the raw data (faded colors) and the filtered signals (solid colors) to demonstrate the effect of data processing and highlight underlying trends.

To conduct HER measurements, the MoS_2_ NRs were transferred onto highly oriented pyrolytic graphite (HOPG) substrate, which serves as a conductive but catalytically inactive substrate. Figure 5A shows an SEM image of two wide MoS_2_ NRs on HOPG, with widths of ~290 and ~250 nm, and that of a narrow NR with a width of ~55 nm. Figure 5B displays the corresponding HER current mapping image for the same area, measured at −0.95 V versus reversible hydrogen electrode (RHE). The HER current mapping of the wider NRs distinctly reveals the current from each edge. The current from the edges is much higher than that from center of the NRs. As shown in the current profile in Fig. 5C, the current from edges is approximately two orders of magnitude higher than that at the center, indicating a superior catalytical activity of the edges.

Figure 5 (D and E) also shows that the edges of the NRs have a lower overpotential and smaller Tafel slope compared to the center of the NR and to the HOPG. The Tafel slope provides insight into the rate-determining step of the HER, with smaller slopes indicating better catalytic performance. Our estimated Tafel slope of 139 mV/dec for the MoS_2_ NR edges suggests that the Volmer reaction is likely the rate-determining step in our system, indicating that hydrogen adsorption is the most favorable reaction for our MoS_2_ NRs. To contextualize our results within the broader field of HER catalysts, we compared our findings with those of other materials (see table S1). The observed wide NRs evidence differences in the current contribution from each edge (see, for example, the rightmost NR in Fig. 5, B and C), suggesting that one edge generates more current than the other. This observation implies inherent disparities in the edge morphology and/or composition between the two edges of a same NR (as discussed in fig. S8) and their impact on HER activity. Current studies are underway to elucidate the specific effects of edge morphology and composition on the HER performance.

Although the SECCM used in this study is capable to resolve features down to several tens of nanometers, it presents a challenge distinguishing between the edges of the narrow NR (~55 nm). This is because the width of the NR is comparable to the diameter of the nanopipette used in the SECCM measurements (~40 nm). Consequently, the HER current response measured on the narrow NR is roughly equal to twice that on each edge of the wide NR as it encompasses the HER current responses from both the left and right edges of the narrow NR. This highlights the efficiency of having dense arrays of narrow NRs, with their high edge-to-area ratio, for increasing the current density in HER applications compared to large 2D flakes.

### Transport properties and performance of MoS_2_ NR FETs

**Fig. 6.**
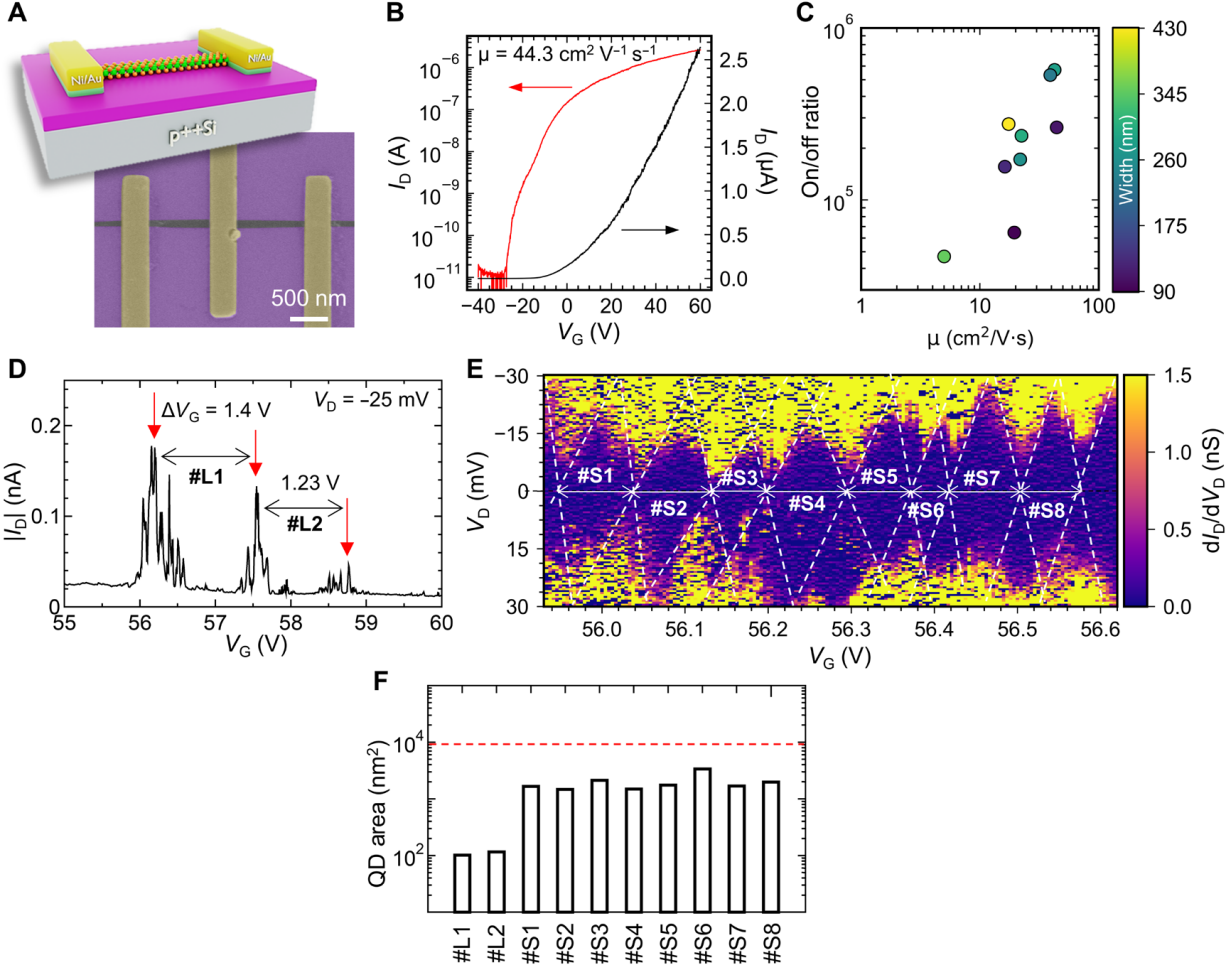
Electrical performance of the MoS_2_ NRs. (**A**) Schematic (top) and false-colored SEM image of an actual NR device (bottom). (**B**) Room temperature transfer characteristics of an FET with an NR channel with a width of 110 nm. (**C**) Mobility versus on/off ratio of several NR devices, collected at room temperature. The width of the NRs is indicated by the color of the markers. (**D**) Long-period Coulomb oscillation peaks of a device with an NR channel with a width of 30 nm measured at 4.2 K. The red arrows indicate the periodicities. (**E**) Edge-highlighted short-period Coulomb diamond features of the 30-nm-wide NR device. (**F**) Comparison of whole NR area (9000 nm^2^, indicated by the red dashed line) and calculated QD sizes from (D) and (E).

The transport properties of the MoS_2_ NRs were evaluated using back-gated FETs, as shown in the SEM image of Fig. 6A. The transfer characteristics of an FET with a 110-nm–wide NR channel is shown in Fig. 6B, displaying a carrier mobility of 44 cm^2^ V^−1^ s^−1^. The device sustains a current density of ~22.7 μA μm^−1^ (3.25 MA cm^−2^) at a bias voltage of 1 V, which is comparable to the values obtained for high-quality exfoliated MoS_2_ flakes (*49*). This high current density, along with the remarkable mobility, underscores the high quality of the MoS_2_ NRs and their potential for applications in high-performance electronic devices (*8*). The linearity of the output characteristics of the device, shown in fig. S20, evidences a good ohmic contact between the NR and the electrodes at room temperature. Figure 6C presents the carrier mobility versus the on/off ratio for devices with different channel widths, represented by the color of the markers. Although there is a positive correlation between the mobility and the on/off ratio, the mobility appears to be independent of the NR width, with an average value of 25.4 cm^2^ V^−1^ s^−1^ at room temperature.

To verify the emergence of 1D characteristics in the MoS_2_ NRs, detailed electrical conductivity measurements were conducted at a low temperature (4.2 K) for an NR with a width and length of 30 and 300 nm, respectively. This device is small enough to behave as a quasi-0D quantum dot (QD). If defect-free MoS_2_ NRs are synthesized, then we can expect to observe Coulomb oscillation with fine oscillation periods, where the entire MoS_2_ NR becomes a single QD. On the other hand, in the case of MoS_2_ NRs with many defects, multiple QDs will be dominant, and it can be expected that only nonperiodic Coulomb oscillation will be observed. In other words, the quality of the MoS_2_ NRs can be inferred from the shape of the observed quantum features. Because the threshold of the n-type current at this low temperature exists around *V*_G_ from 55 to 60 V (fig. S21A), the *V*_D_-*V*_G_ dependence of the differential conductance ($dI_{D}/dV_{D}$) was measured in this region. As a result, large periodic Coulomb oscillations with peak intervals Δ*V*_G_ ranging from 1.23 to 1.4 V (#L1 and #L2) (Fig. 6D) and Coulomb diamonds (fig. S21B) were observed. Closer examination of a single Coulomb oscillation, specifically within the *V*_G_ range of 56 to 57 V, uncovered the presence of finer periodic oscillation components (fig. S21C). Detailed measurements in this expanded region revealed the existence of eight highly periodic Coulomb diamonds (#S1 to #S8), as shown in Fig. 6E.

The observation of Coulomb diamonds with both long (#L1 and #L2) and short (#S1 to #S8) periods indicates the presence of QD-like structures and QDs of different sizes within the synthesized NR. Although it is difficult to completely identify the QD sizes due to the presence of multiple structures, order estimation of QD size can be possible from these periodic peaks to infer the origins of the large (#L1 and #L2) and small (#S1 to #S8) periodic components. The QD size was calculated based on the general planar model (*50*). Because the width of the NR (30 nm) is sufficiently small compared to the SiO_2_ oxide film (300 nm), the electric field concentration effect was also taken into account in the calculations (*51*). The results showed that the areas for #L1 and #L2 are 100 to 116 nm^2^, whereas those for #S1 to #S8 are ~1470 to 3390 nm^2^ (Fig. 6F). If we consider the entire NR (with dimensions of 30 nm by 300 nm) acting as a single QD, then the QD area would be ~9000 nm^2^. The QD area obtained for #S1 to #S8 corresponds to about 16.3 to 37.6% of the entire MoS_2_ NR, suggesting the formation of relatively large QDs across the entire MoS_2_ NR. Considering edge roughness and the Schottky barrier with the electrodes, it is expected that the effective QD area is smaller than the geometrically calculated area (9000 nm^2^). Therefore, this explanation of the entire MoS_2_ NR structure acting as a single QD may be reasonable by considering demonstrated the high quality of the MoS_2_ NRs (Fig. 2, A to C). On the other hand, the long-period components (#L1 and #L2) correspond to QD-like structures with a diameter of about 10 nm and are likely due to minor local defects or edge roughness present in the MoS_2_ NR.

## DISCUSSION

In this work, we demonstrate the lattice-guided mode for the self-aligned growth of TMD NRs on low-symmetry substrates, such as a-plane sapphire. DFT calculations revealed that this method is enabled by the anisotropy in the precursor diffusion on the a-plane sapphire that results in the alignment of the NRs along the $\left[1\bar{1}00\right]$ direction of sapphire. By increasing the growth temperature up to 1100°C, the shape of the MoS_2_ evolved from the normal triangular flake shape to NRs with widths below 10 nm. SHG measurements and optical and STEM images revealed that the longitudinal axis of the NRs is parallel to the MoS_2_ ZZ direction. We showed that our method can be extended to the growth of lateral NR heterostructures by varying the precursors during the growth process. Furthermore, the edges of the MoS_2_ NRs showed a high HER current response, which, besides their high edge-to-area ratio, makes them promising candidates for efficient catalysis applications. Both STEM and transport property analysis evidenced the high quality of the NRs, highlighting their potential for future electronic and optoelectronic devices.

## MATERIALS AND METHODS

### Synthesis of the NRs

The growth of the TMD NRs was conducted in a four-zone furnace with a 26-mm–inner diameter quartz tube at ambient pressure with a flow of 300 SCCM (standard cubic centimeter per minute) of Ar as the carrying gas (fig. S12). Sulfur (Mitsuwa Chemical Co., 99.999%, 120 mg) and MoO_3_ (Wako Pure Chemical Industries, 99.9%, 15 mg) powder precursors were located upstream and heated at 200° and 600°C, respectively. For the growth of WS_2_, the WO_3_ precursor was heated at 1060°C. The a-plane sapphire substrate (Kyocera Co.) used had a small miscut angle (<0.3°) off the ideal $\left(11\bar{2}0\right)$ plane, resulting in the formation of step edges that are determined by the magnitude and direction of the miscut angle, rather than following a specific crystallographic direction of the sapphire. The sapphire was cleaned by sonication in acetone and isopropanol prior to the CVD growth. Additional experiments were also performed using r-plane sapphire (Kyocera Co.). For the effective growth of NRs, we found that limiting the amount of precursors is a critical factor. For this, the sapphire substrate was located downstream, rather than face-down above the MoO_3_ precursor (see fig. S12). The growth was performed by heating the sapphire at temperatures ranging from 750° to 1200°C, with 1100°C being the optimal temperature for obtaining narrow NRs. The growth duration at the target temperature was 10 min, followed by cooling of the furnace.

For the growth of the MoS_2_-WS_2_ hNRs, we used a two-step process to avoid the mixing of the precursors. MoS_2_ NRs were first produced by the method explained above (MoO_3_ and sulfur precursors at 600° and 200°C, respectively, with the substrate at 1100°C). The as-grown MoS_2_ NRs were then transferred to a separate, dedicated furnace for the WS_2_ growth (WO_3_ and sulfur precursors at 1060° and 200°C, respectively, and the substrate at 1100°C).

### Transfer of the NRs

The transfer of the NRs to SiO_2_ and HOPG substrates was performed by a wet-transfer method. First, polymethyl methacrylate (PMMA) was spin coated on the as-grown NRs. The PMMA with the NRs was released from the sapphire by etching in a 1 M KOH aqueous solution for up to 10 min. The detached PMMA with the NRs was washed in ultrapure water and deposited in the target substrate (SiO_2_ or HOPG), which was then heated to 125°C. Afterward, the PMMA was removed in hot acetone (60°C) for 8 hours and dried with a stream of N_2_.

Graphene-coated Quantifoil TEM grids were used to support the narrower NRs for STEM measurements. SLG was obtained by CVD on Cu thin films sputtered on c-sapphire (*52*) and transferred to Quantifoil TEM grids without the use of polymers. The SLG/TEM grid was then attached to the as-grown NRs and immersed in KOH to detach the NRs from the sapphire substrate. After detachment, the NRs/SLG/TEM grid was washed in ultrapure water.

### Characterization

The optical characterization of the samples included confocal Raman and PL spectroscopies, performed at room temperature using a Nanofinder 30 spectrometer (Tokyo Instruments) with a 532-nm laser excitation and a power of 0.1 mW. SHG measurements were conducted using a backscattering geometry with a pulsed laser (900-nm wavelength, 100-fs pulse width, and 80-MHz repetition rate) and a spot size of ~1 μm. The reflected SHG signal from the sample was collected by the same objective and passed through a series of optical components to a thermoelectrically cooled high-efficiency complementary metal-oxide semiconductor (CMOS) detector. Detection was performed without a monochromator, allowing for the capture of small signals at the expense of wavelength-resolved measurement.

Microscopical inspections included SEM (Hitachi S-4800) and AFM (Bruker Nanoscope V) to characterize the NRs. STEM and EELS analyses were performed at room temperature using a JEOL-tripleC#3 ultrahigh vacuum microscope (JEOL-ARM200F based) equipped with a JEOL delta corrector and a cold field-emission gun operating at 60 kV. EELS core loss spectra were collected by using the Gatan GIF Continuum with Rio CMOS camera optimized for low-voltage operation. The STEM simulated images were generated using the software Dr. Probe (*53*).

### DFT calculations

All calculations were based on DFT (*54*) as implemented in the STATE (Simulation Tool for Atom TEchnology) program package (*55*). We used the vdW-DF2 with the C09 functional to describe the exchange-correlation potential energy among the interacting electrons, which enabled the investigation of the weak dispersive interaction between the Al_2_O_3_ surfaces and MoS_2_ flakes (*56*). Ultrasoft pseudopotentials generated by the Vanderbilt scheme were used to describe the interaction between electrons and nuclei (*57*). The valence wave functions and deficit charge density were expanded using plane wave basis sets with cutoff energies of 25 and 225 Ry (rydberg), respectively. The a-plane Al_2_O_3_ surfaces are simulated by a repeated slab model with eight O atomic layers with a rectangle of *a* = 2.598 nm and *b* = 1.647 nm. The MoS_2_ flake is simulated by the smallest trapezoid comprising 10 Mo and 18 S atoms. The atomic structures were fully optimized until the force acting on each atom was less than 1.33 × 10^−3^ hartree/bohr. We used the effective screening medium method to investigate the electronic and electrostatic properties of thin films within the conventional DFT framework (*58*).

### HER measurement

The details of the SECCM setup have been previously reported (*48*). Briefly, a movable nanopipette (tip diameter of 40 nm) containing a 0.5 M aqueous H_2_SO_4_ electrolyte and an Ag/AgCl quasi-reference counter electrode (QRCE) were used. The applied potential was −0.95 V versus RHE. Potentials were converted to RHE from Ag/AgCl QRCE. The nanopipette was moved to the sample surface at 10 nm/ms using a z-piezo stage and stopped upon detecting a capacitive current (0.3-pA threshold). The redox current was measured after 2 ms with a measurement time of 50 μs. After each measurement, the nanopipette was retracted by 1.0 μm and the sample was moved to the next point in the *xy* plane. By repeating this process, topographic and current images were acquired simultaneously. Local cyclic voltammetry (CV) measurements were performed by selecting specific locations in the SECCM image and at a sweep rate of 5.0 V/s.

### FET fabrication and measurement

To assess the transport properties of the NRs, back-gate MoS_2_ NRs FETs were fabricated on SiO_2_ (300 nm)/Si. Electron-beam lithography using a double-layer resist of PMMA 495K and PMMA 950K (both 6% by weight in anisole, spin coated sequentially at 4000 rpm) was used to define the source and drain electrodes, followed by the deposition of Au/Ni (30 nm/3 nm) electrodes by electron-beam evaporation. Room temperature measurements were carried out in vacuum (~10^−4^ Pa) using a Keysight B1500A semiconductor analyzer. The low-temperature (~4.2 K) electrical measurements of the MoS_2_ NR devices were performed with a cryogenic measurement system. The devices were mounted on a dipstick and cooled down by liquid helium. The currents were measured using a semiconductor test system (HP 4142B modular dc source/monitor). The field-effect mobilities were calculated from the equation $\mu_{\text{FE}}=g_{m}\frac{L}{WC_{\text{ox}}V_{d}}$ (*52*), in which *L* and *W* are the channel length and width, respectively, *C*_ox_ is the gate capacitance per unit area, and *V*_d_ is the drain voltage. The value of the transconductance, $g_{m}=\frac{\partial I_{d}}{\partial V_{g}}$, was obtained from the linear region of the transfer curves.

## Supplementary Materials

### The PDF file includes:

Figs. S1 to S21 Table S1 Legend for movie S1 References
