# Supplementary material for: Lattice-guided growth of dense arrays of aligned transition metal dichalcogenide nanoribbons with high catalytic reactivity
Source: Sci Adv. 2025 Jan 8;11(2):eadr8046. doi: 10.1126/sciadv.adr8046 (PMC11708881; doi:10.1126/sciadv.adr8046)
Supplement: Supplementary file 1 — Figs. S1 to S21 Table S1 Legend for movie S1 References [file sciadv.adr8046_sm.pdf]

Science Advances
AAAS

Supplementary Materials for

Lattice-guided growth of dense arrays of aligned transition metal dichalcogenide nanoribbons with high catalytic reactivity

Zongpeng Ma *et al.*

Corresponding author: Pablo Solís-Fernández, solisfernandez.pablo.kyudai@gmail.com; Hiroki Ago, ago.hiroki.974@m.kyushu-u.ac.jp

Sci. Adv. **11**, eadr8046 (2025)
DOI: 10.1126/sciadv.adr8046

The PDF file includes:

Figs. S1 to S21
Table S1
Legend for movie S1
References

Other Supplementary Material for this manuscript includes the following:

Movie S1

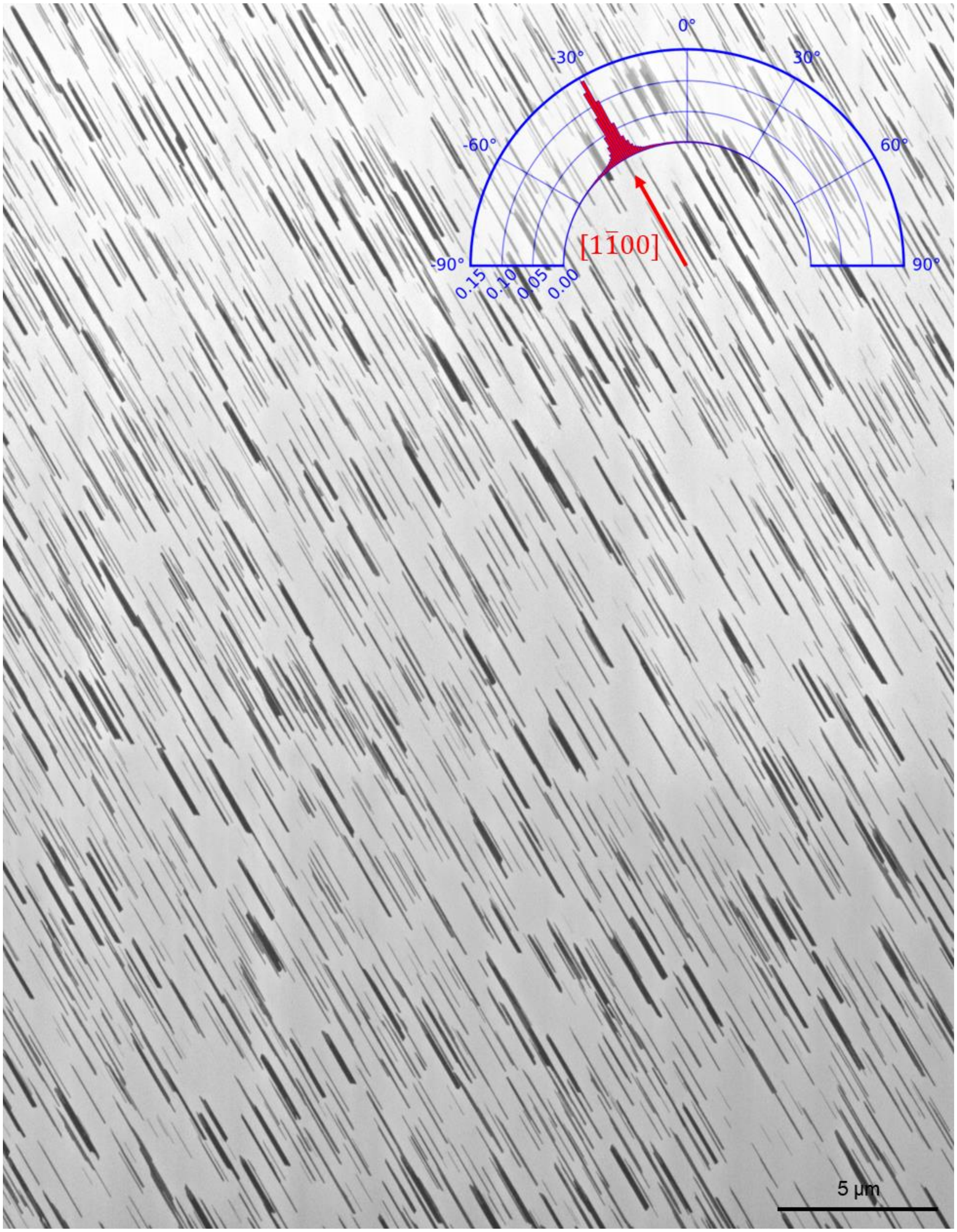

Fig. S1.

Stitched SEM image of MoS_2 nanoribbons on a-plane sapphire. The inset shows the histogram in polar coordinates of the alignment of the ribbons in the area, evidencing a narrow distribution parallel to the $[1\bar{1}00]$ direction of sapphire indicated by the red arrow.

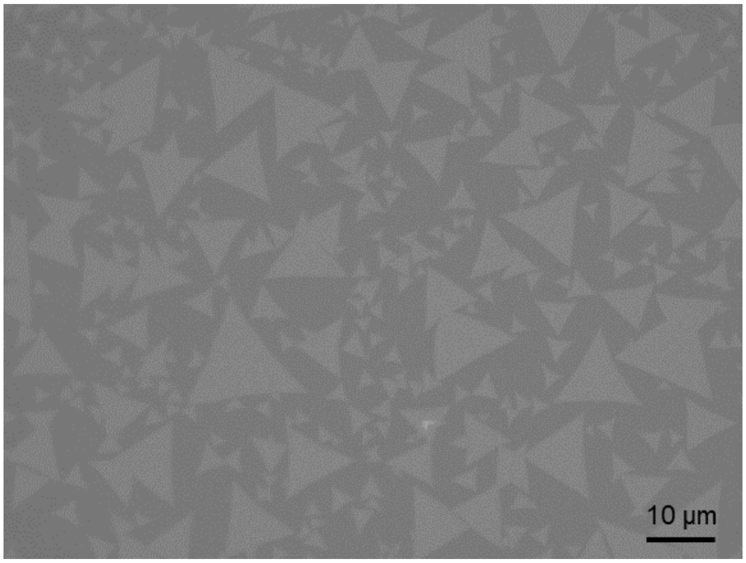

Fig. S2.

Optical microscope image of MoS_2 grown on c-plane sapphire using the same CVD growth condition (1100 °C) as for the sample shown in Fig. 1B. Triangular flakes were obtained on the c-plane sapphire instead of the oriented NRs obtained on a-sapphire.

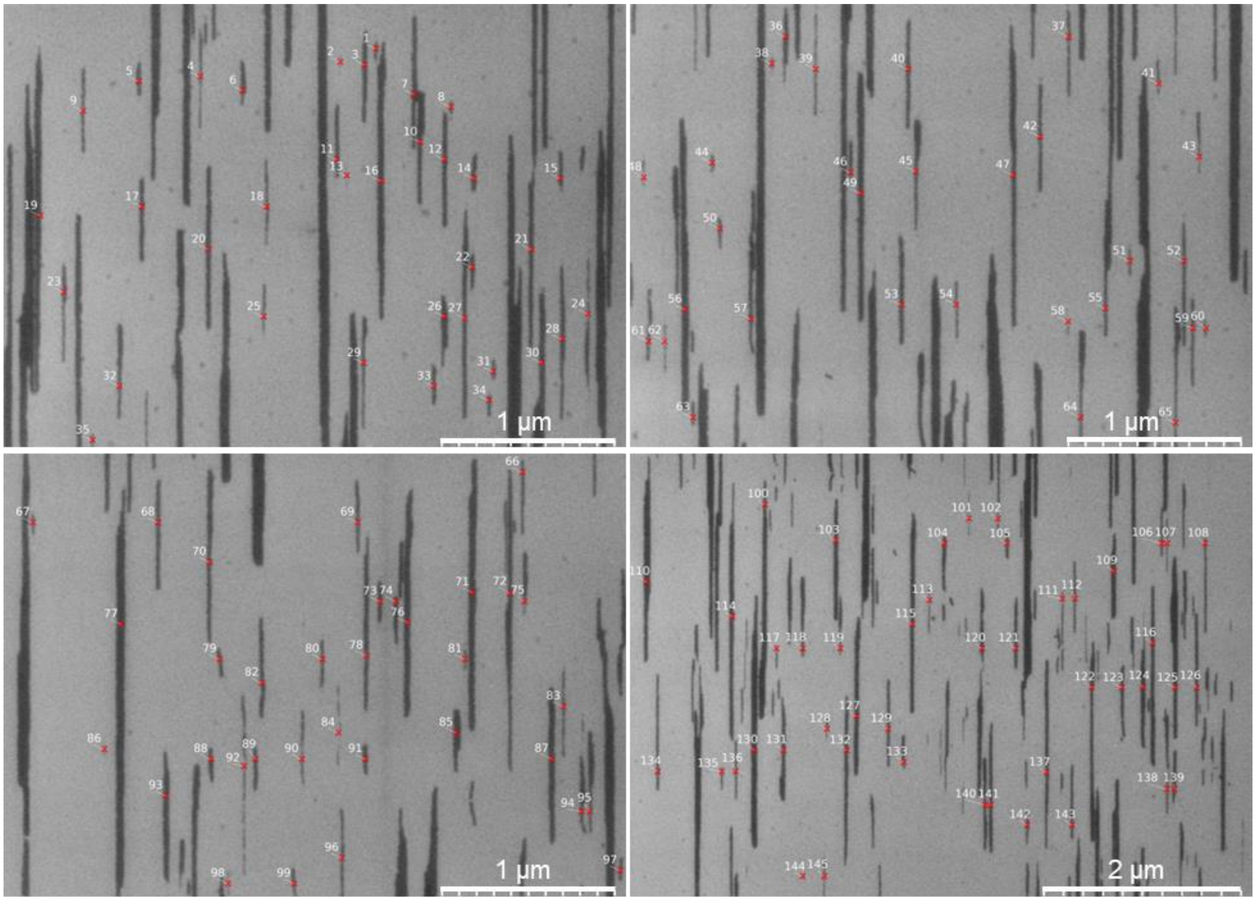

Fig. S3.

SEM image showing the MoS_2 NRs from which statistics used in Fig. 1C were collected.

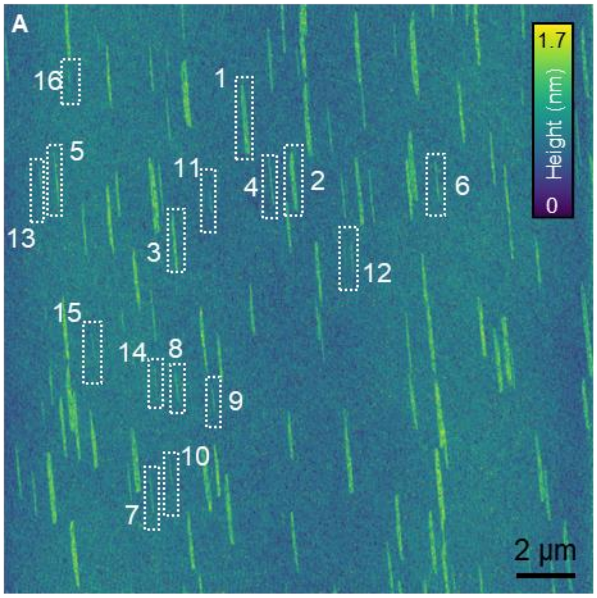

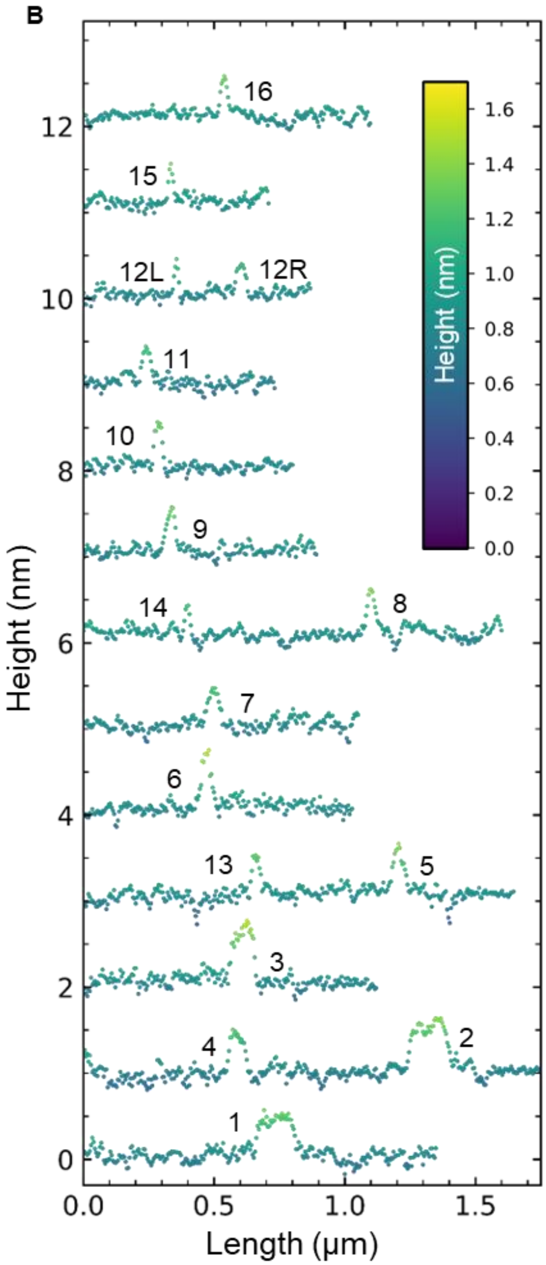

C

Ribbon #	Width (nm)	Height (Å)
1	147.1	4.1
2	151.5	5.2
3	92.9	5.8
4	68.4	4.6
5	48.8	5.0
6	58.8	6.1
7	58.7	4.0
8	39.2	4.8
9	44.1	4.9
10	34.1	4.9
11	39.0	3.9
12 (left)	24.4	4.1
12 (right)	48.8	3.4
13	34.2	4.0
14	24.5	3.3
15	24.6	4.0
16	29.4	4.2

Fig. S4.

Size of the MoS_2 nanoribbons by AFM. (**A**) Large-area AFM image of the NRs shown in Fig. 1D, E. The selected NRs are highlighted by white dotted rectangles. (**B**) Height profiles across each of the NR highlighted in (A). The profiles were vertically shifted for clarity, and the color indicates the actual height. (**C**) Measured values of width and height of each of the NRs of (A) and (B). The average height of the NRs is ~4.5 Å, indicating their single-layer nature.

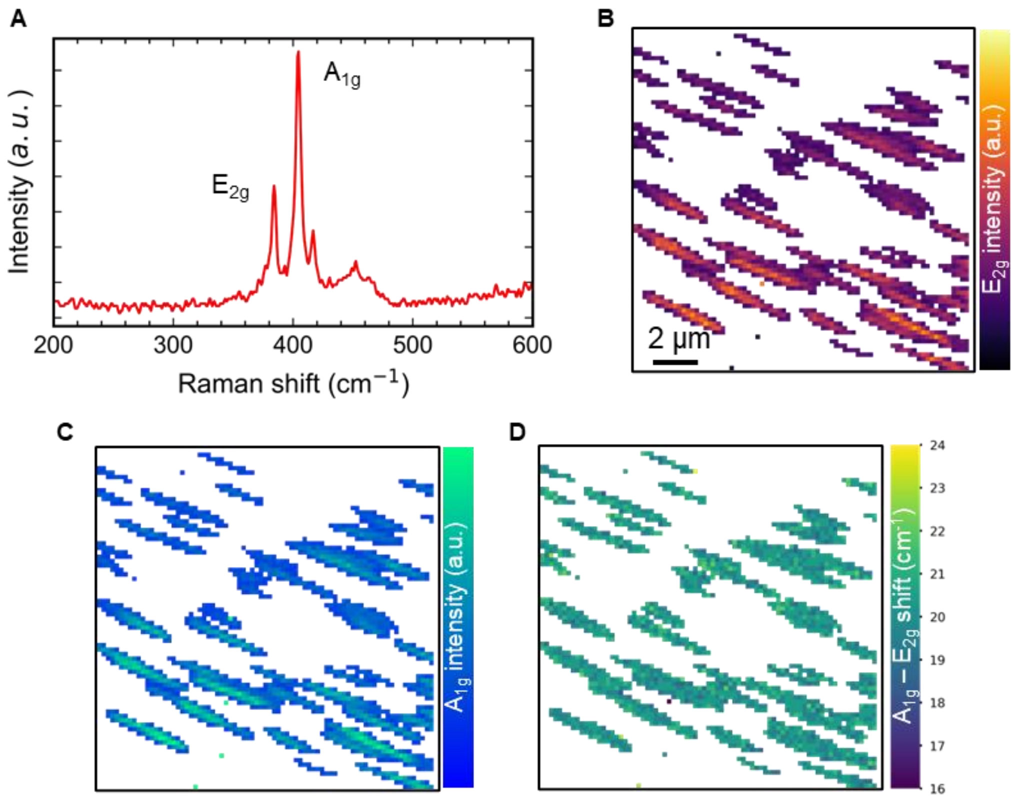

Fig. S5.

Raman characterization of the MoS_2 nanoribbons. (**A**) Raman spectrum of a MoS_2 NR. (**B-D**) Raman mappings of the E_{2g} (**B**) and A_{1g} (**C**) intensity, and of the (A_{1g} – E_{2g}) shift difference (**D**) of MoS_2 NRs on a-sapphire. The uniform peak distance in the Raman spectrum (~20 cm^{-1}) in (D) is an indicator of the single layer nature of the NRs.

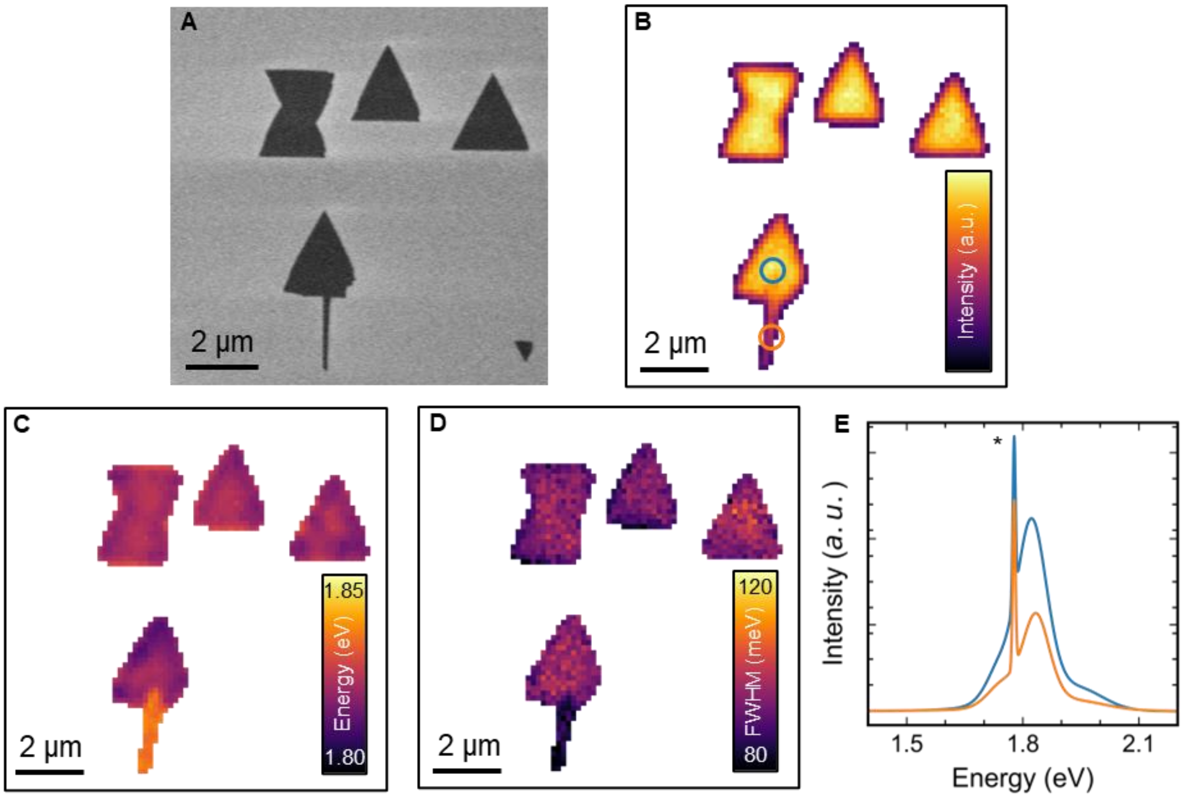

Fig. S6.

Characterization of MoS_2 flakes. (**A-D**) SEM image (**A**) and the corresponding PL mappings for the intensity (**B**), energy (**C**), and full-width at half maximum (FWHM) (**D**) of the A-exciton peak (~1.8 eV) of MoS_2 in an area containing triangular flakes and a NR. (**E**) PL spectra collected at the positions marked in (B), corresponding to a triangle (blue) and a NR (orange). The asterisk in (E) indicates the signal from the sapphire substrate.

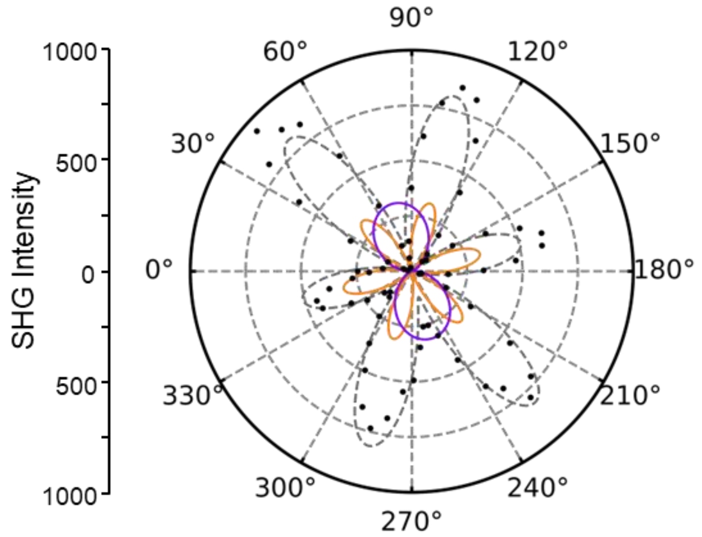

Fig. S7.

Polar plot of the polarization-resolved SHG signal intensity with the individual $\cos^2$ components used in the fitting, with 6-fold (orange) and 2-fold (purple) symmetries. The fit result is indicated by the dashed gray line, and the black dots represent the original data. For clarity, the individual components have undergone baseline correction to shift the minimum intensity to zero.

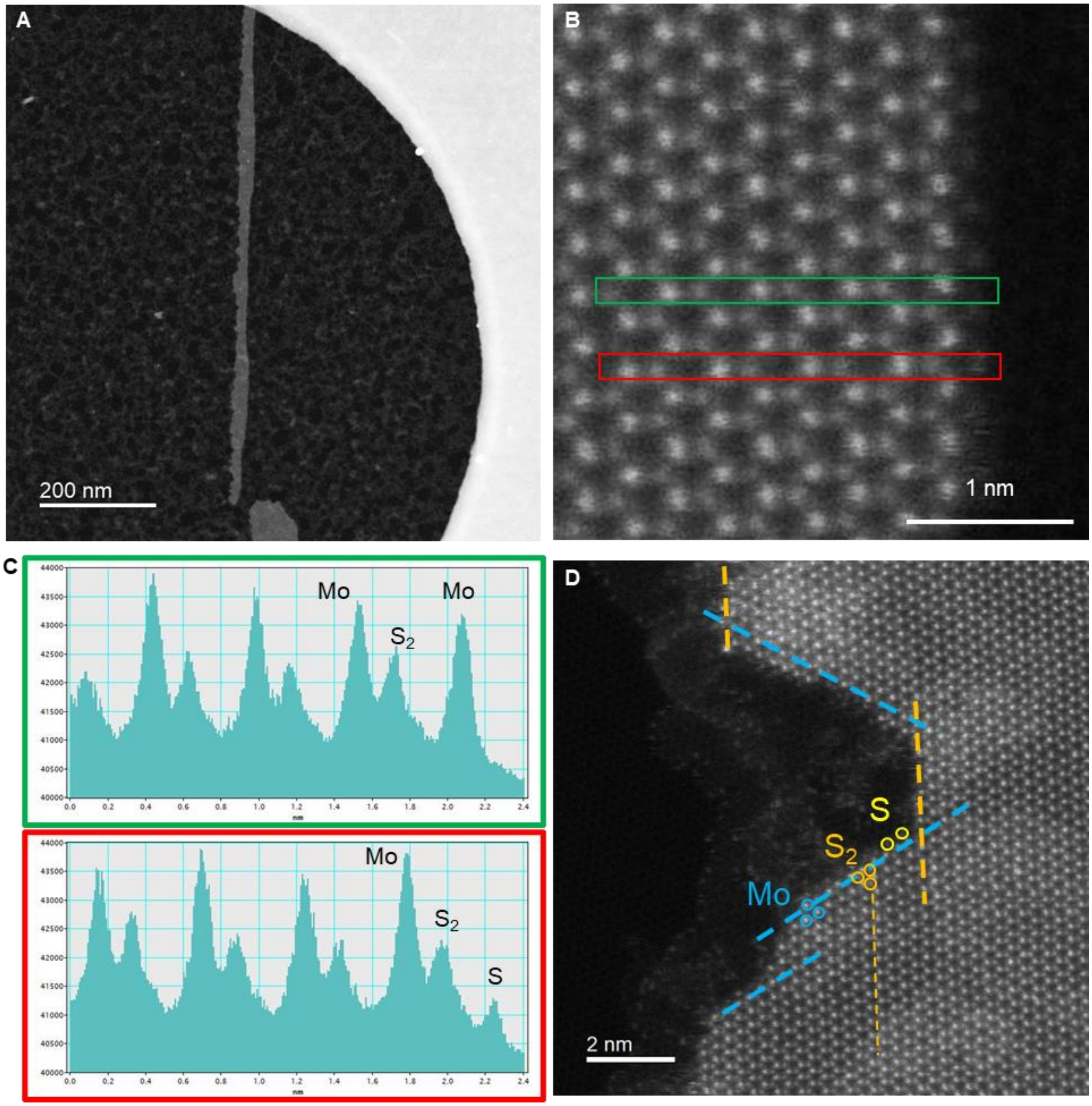

Fig. S8.

Side-dependent edge structure of the MoS_2 nanoribbons. (**A**) General STEM image of a MoS_2 NR supported on SLG, showing the difference between the ZZ-S_2 edge (left) and the ZZ-Mo edge (right). (**B**) Atomic scale resolution STEM image of the ZZ-Mo edge. (**C**) Intensity profiles collected along the indicated areas in (B), showing the termination with a single S atom of the Mo atoms of the edge (ZZ-Mo(S)). (**D**) High resolution STEM image of the ZZ-S_2 edge. The edge retains a global direction parallel to the ZZ direction of the MoS_2, but it is composed by a mixture of ZZ-S_2 (indicated by orange dashed lines) and ZZ-Mo(S) (indicated by blue dashed lines) sections that form angles of 60°. Two of the single S atoms in the ZZ-Mo(S) edge are highlighted in yellow.

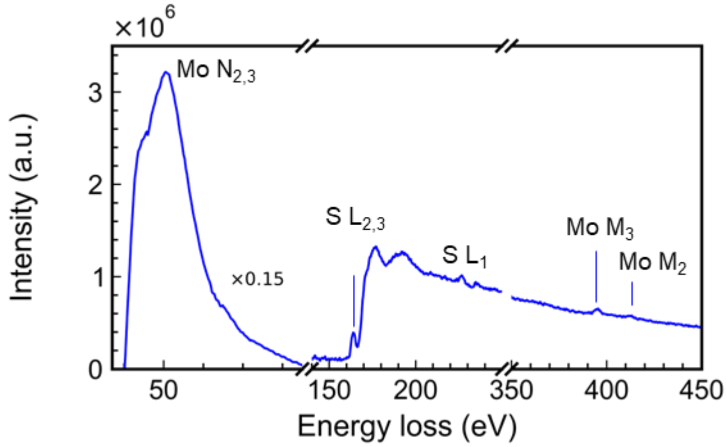

Fig. S9.
EELS spectrum of a MoS_2 NR, showing the S and Mo edges.

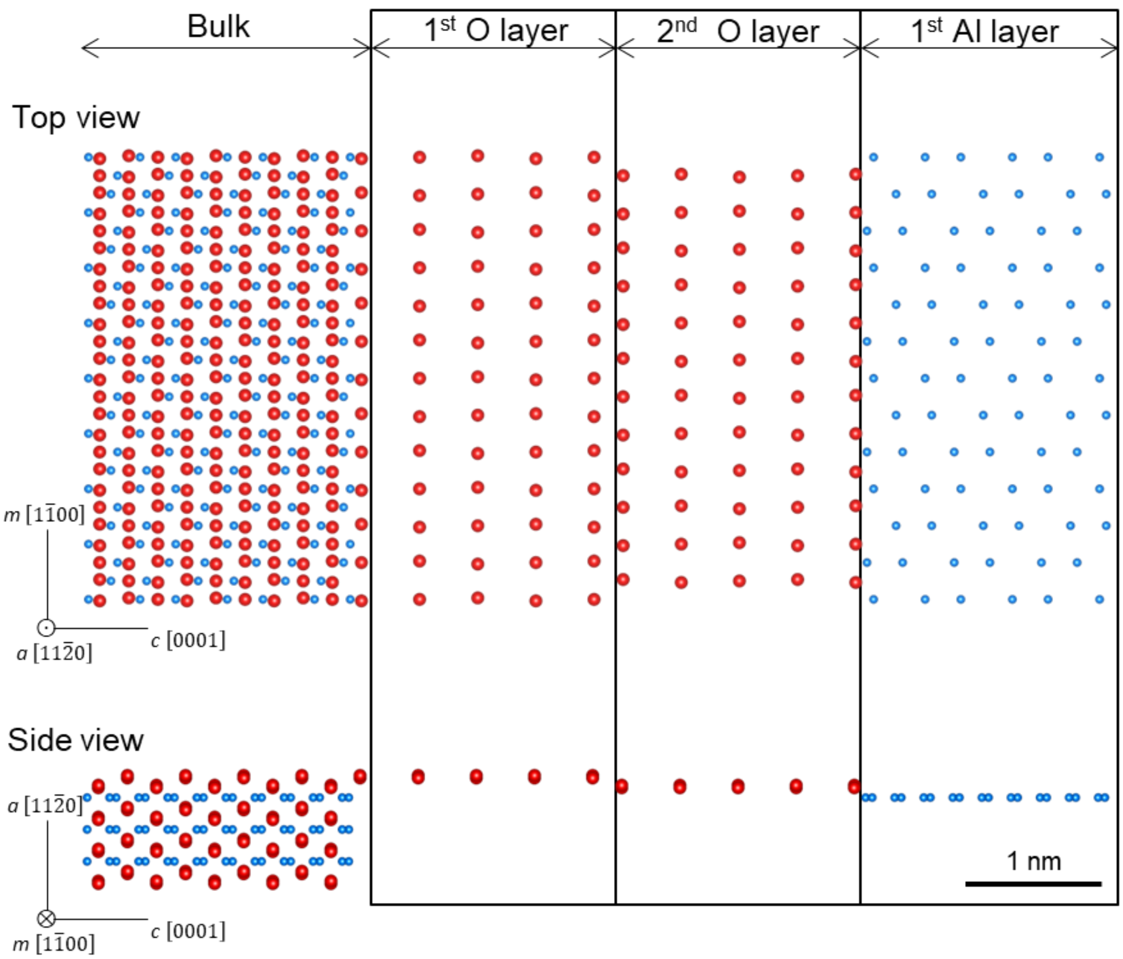

Fig. S10.

Crystal structure of the a-plane sapphire $(11\bar{2}0)$ viewed from the top and from the side (m-plane direction $[1\bar{1}00]$). The figure includes the bulk crystal structure (left side), and that of the top three atomic layers of the surface, corresponding to layers of O, O and Al atoms, respectively. Red and blue atoms represent O and Al, respectively. Linear arrays of O atoms are clearly seen along the $[1\bar{1}00]$ direction.

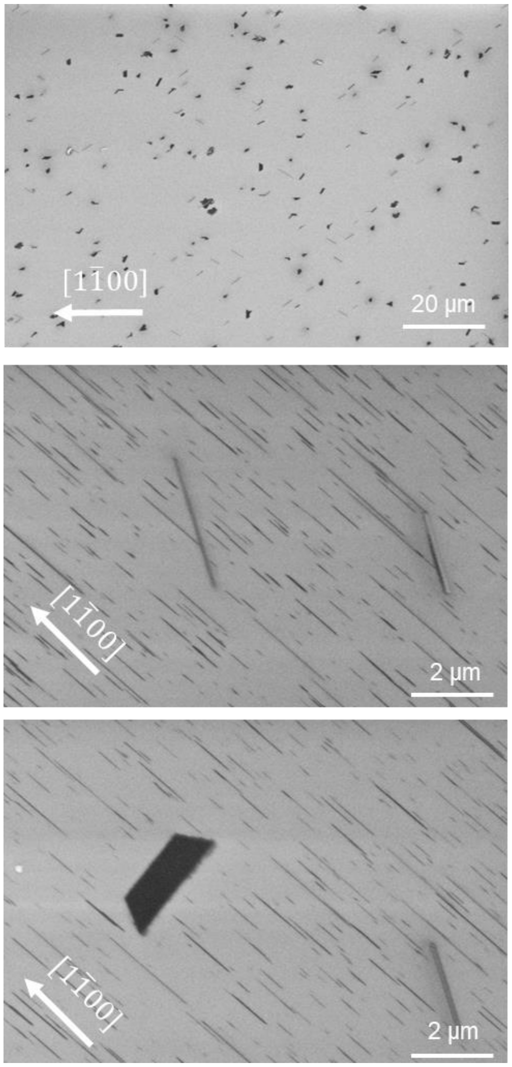

Fig. S11.

SEM images of different regions of the a-plane sapphire after the MoS_2 growth at 1200 °C. The images show the presence of byproducts on the surface, and the misalignment of some of the NRs.

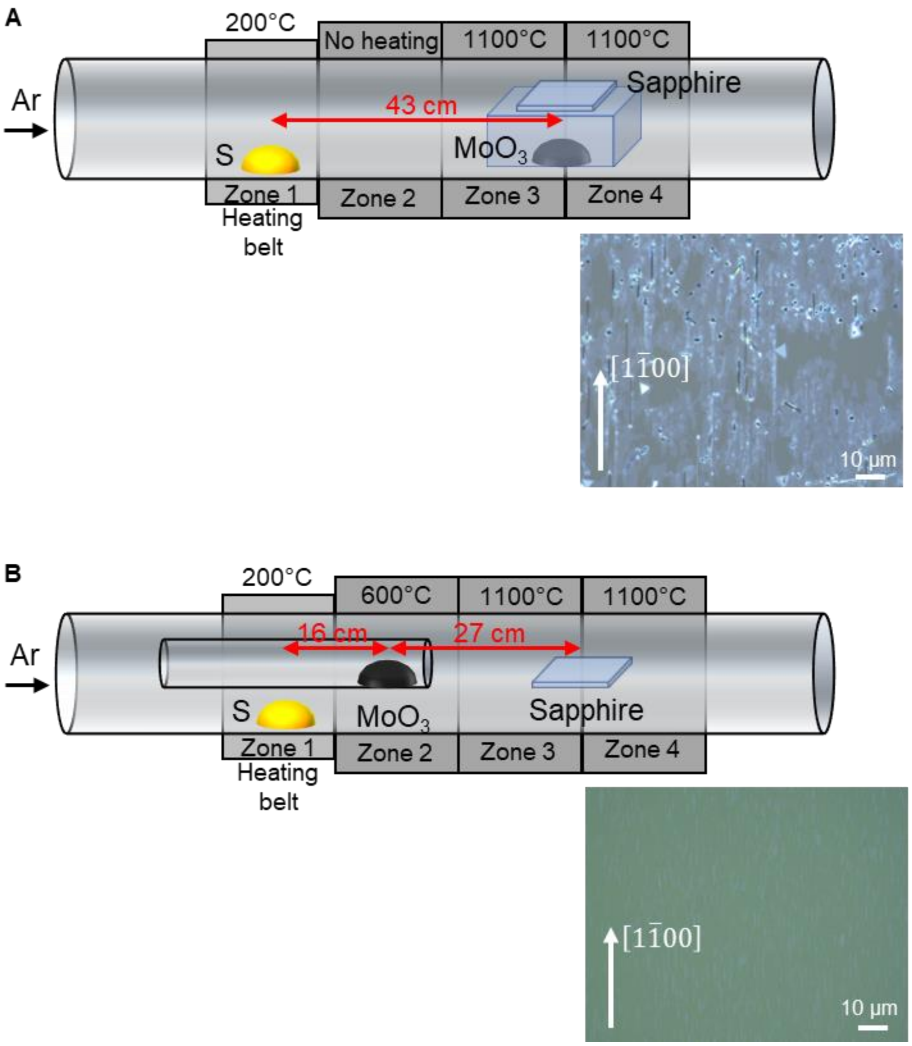

Fig. S12.

CVD setup for the growth of nanoribbons. (**A-B**) Comparison of the face-down (**A**) and downstream (**B**) CVD setups, along with representative optical images of the respective growth at 1100 °C. The face-down setup (A) provides large amounts of precursors when the CVD temperature is increased, making difficult to control the growth of NRs and producing the growth of thick materials. Placing the substrate downstream of the precursors (B) limits the amount of MoO_x and allows for the growth of narrow NRs. Moreover, the MoO_3 was located inside a narrow quartz tube to avoid the sulfurization of the MoO_3 before being evaporated. The distances between the precursors and the substrates are indicated by red arrows, while the sizes of zones 2 to 4 of the furnace were 18 cm.

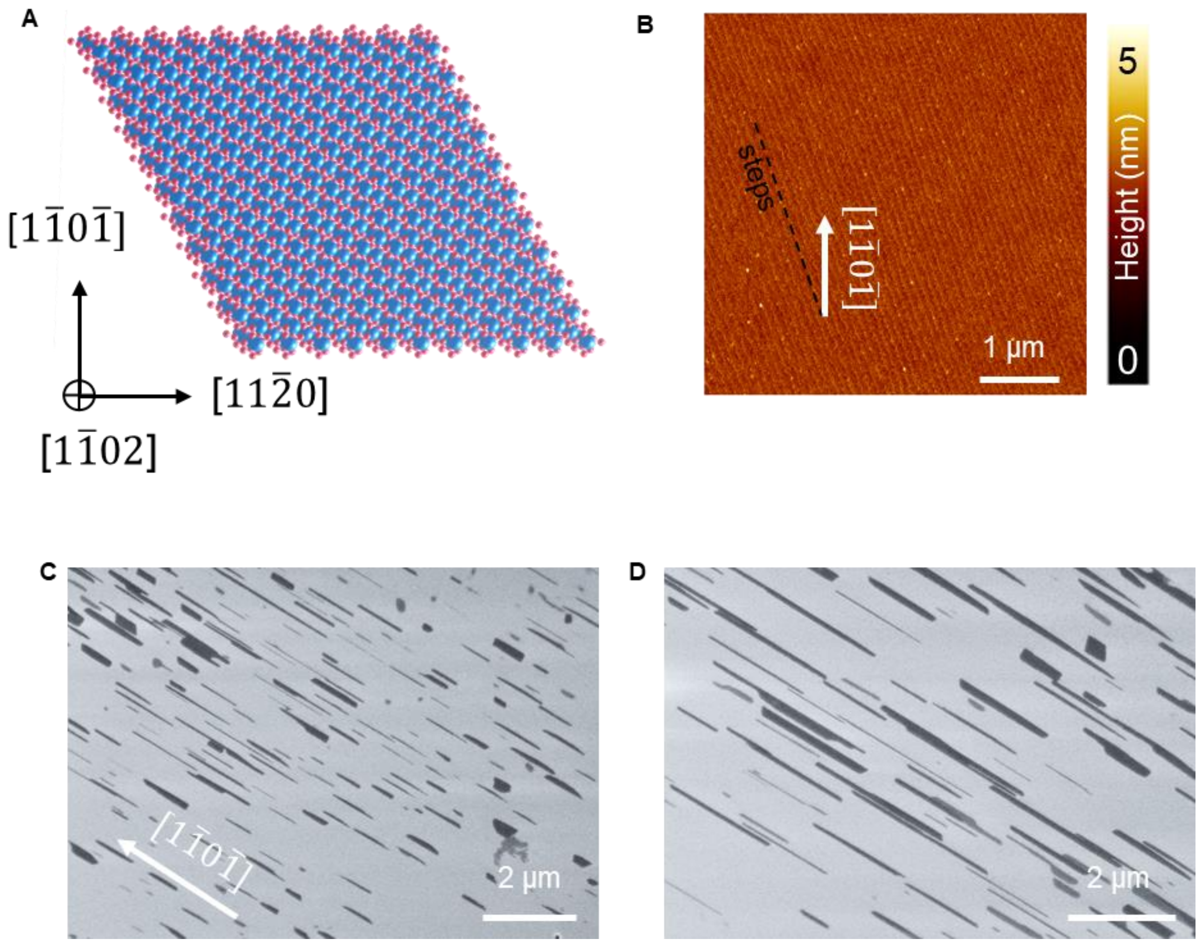

Fig. S13.

Growth of MoS_2 NRs on r-plane sapphire $(1\bar{1}02)$. (**A**) Crystal lattice of the r-plane of sapphire. (**B**) AFM image of the surface of r-sapphire, showing the presence of atomic steps. The steps are not parallel to the $[1\bar{1}0\bar{1}]$ direction of the sapphire. (**C-D**) SEM images showing the presence of MoS_2 NRs on the r-plane sapphire, oriented along the $[1\bar{1}0\bar{1}]$ direction.

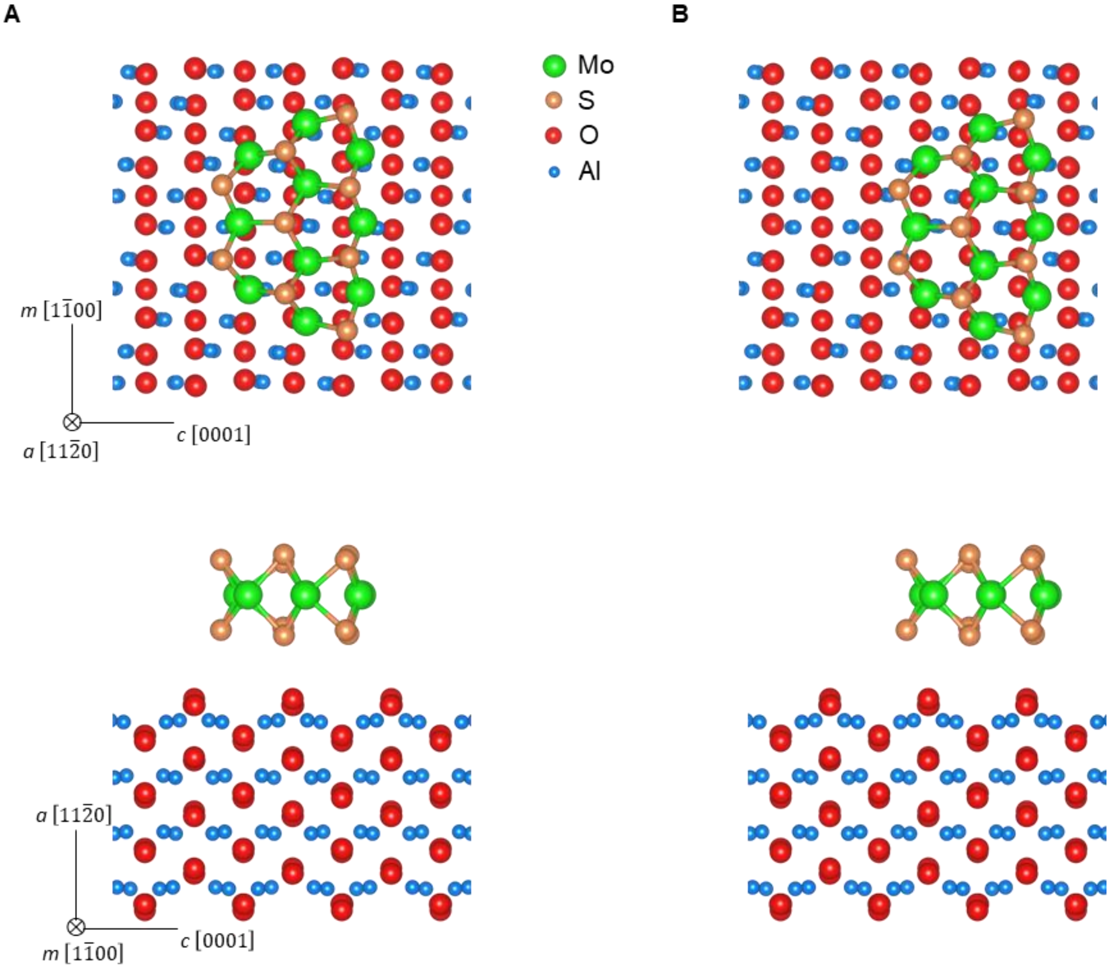

Fig. S14.

Atomic models for the most and least stable DFT configurations. (**A-B**) Atomic models used for the DFT calculations for the most (**A**) and the least (**B**) stable configurations of the MoS_2 on the a-plane sapphire. The models are seen from the $[11\bar{2}0]$ (top) and $[1\bar{1}00]$ (bottom) directions of the sapphire lattice, and include the whole sapphire slab used in the calculations.

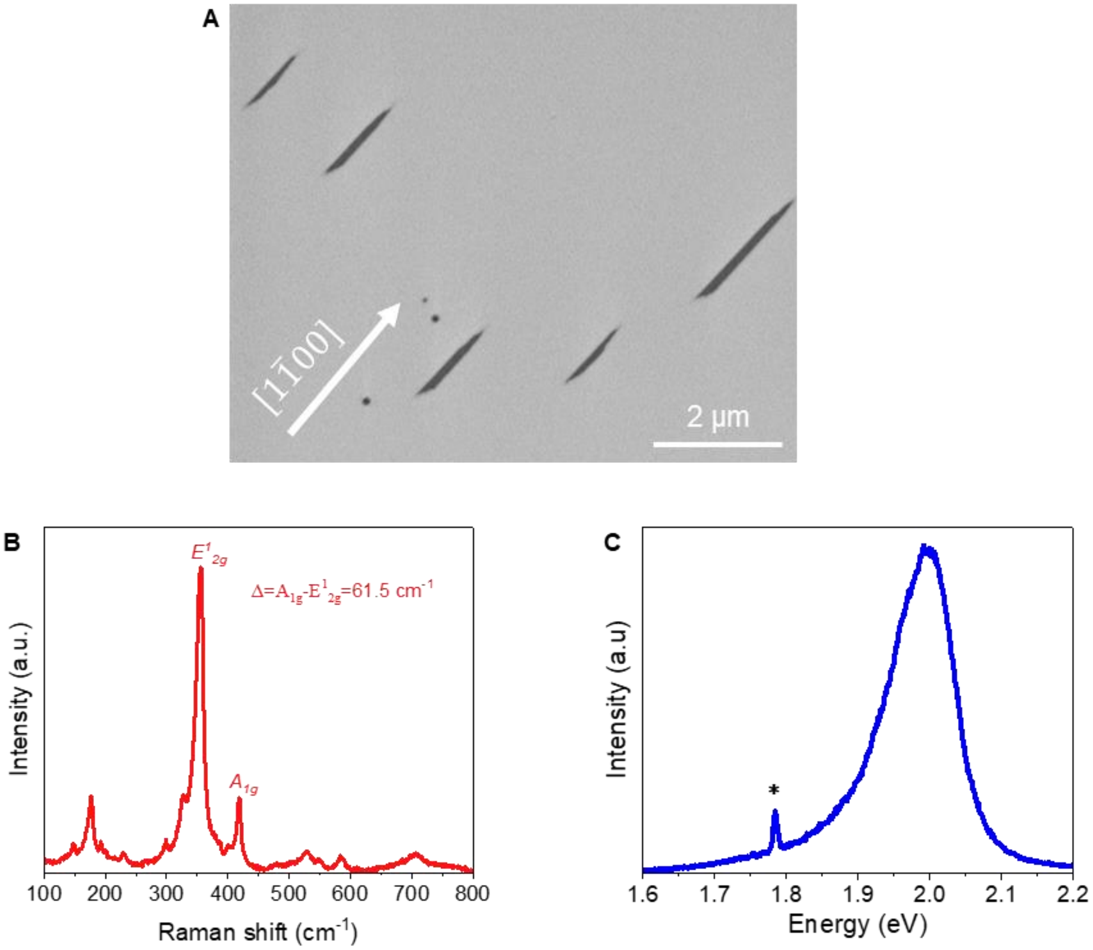

Fig. S15.

Optical characterization of WS_2 nanoribbons. (**A-C**) SEM image (**A**), Raman (**B**) and PL (**C**) spectra of WS_2 NRs grown on a-plane sapphire by changing the precursor from MoO_3 to WO_3. The signal from the sapphire is marked with an asterisk in (C).

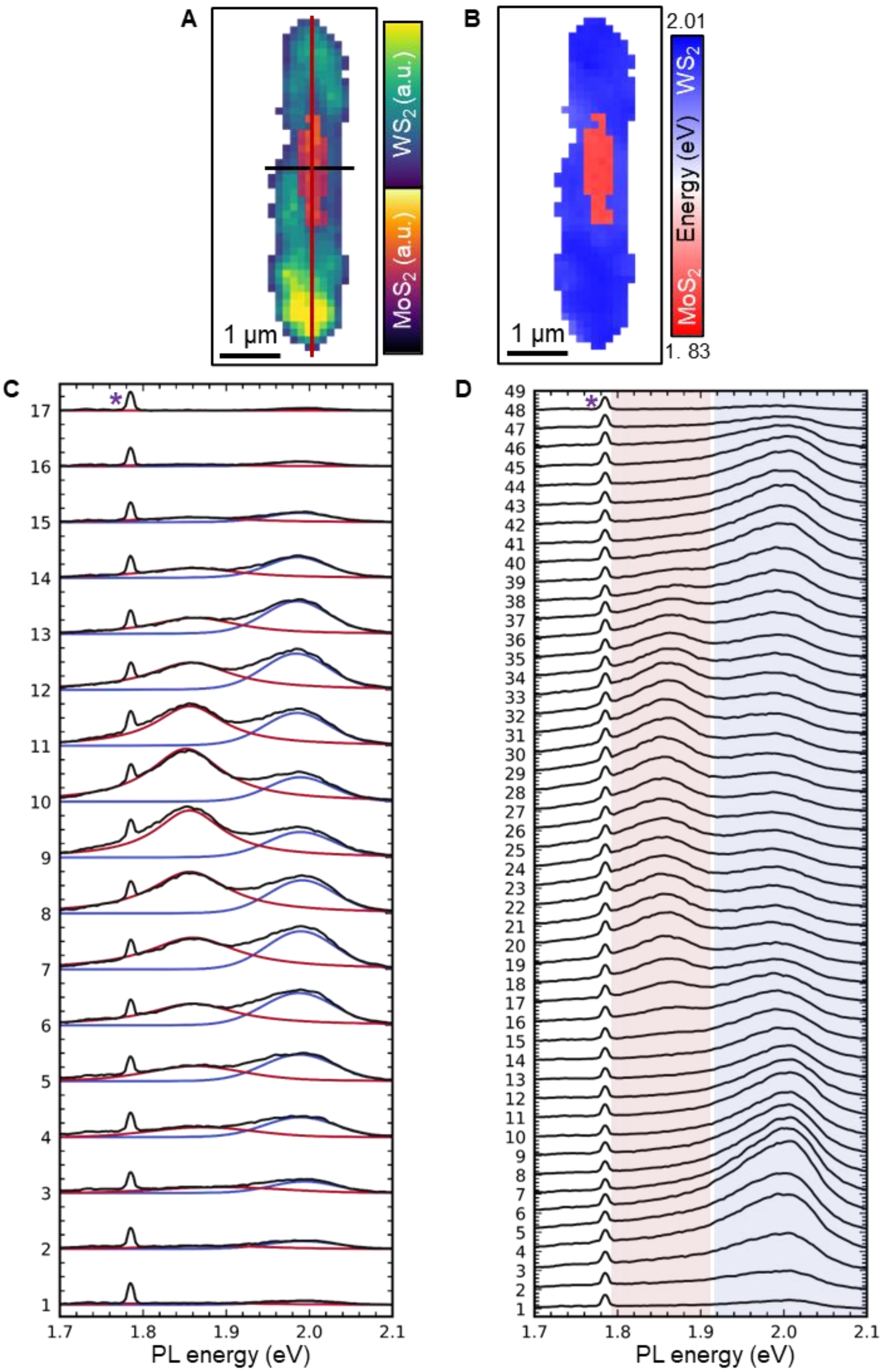

Fig. S16.

PL characterization of MoS_2-WS_2 hetero-nanoribbons. (**A-B**) PL intensity (**A**) and energy (**B**) mapping images of a MoS_2-WS_2 hetero-nanoribbon. (**C-D**) Spectra collected along the horizonal (**C**) and vertical (**D**) lines indicated in (A). The spectra in (C) include the original spectra (black), and the fitted components for MoS_2 (red) and WS_2 (blue). For clarity, only the original spectra are shown in (D). Spectra in both (C) and (D) were collected at regular intervals of 100 nm along the corresponding lines in (A). The peak highlighted by the asterisk in (C) and (D) originates from the sapphire substrate.

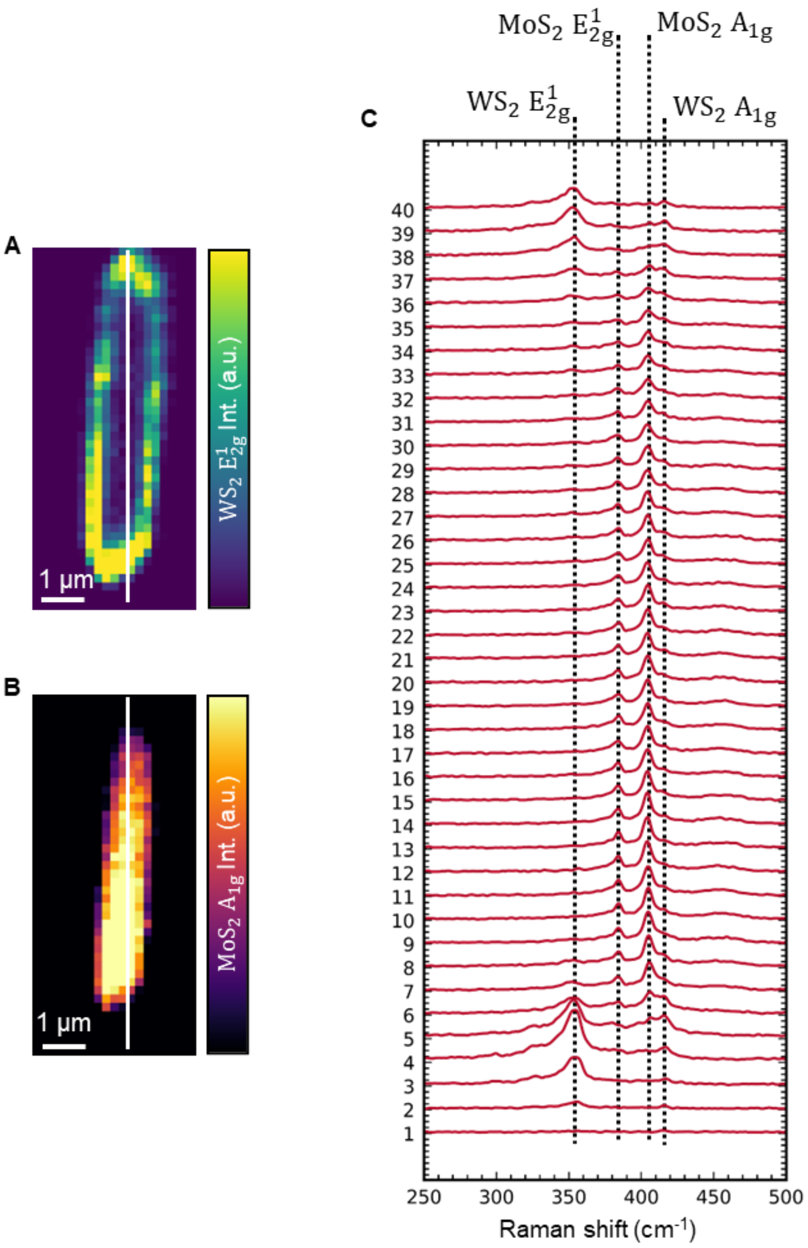

Fig. S17.
Raman characterization of MoS_2-WS_2 hetero-nanoribbons. (**A-B**) Mappings of the intensity of the (**A**) WS_2 E^1_{2g} and (**B**) MoS_2 A_{1g} Raman peaks for a MoS_2-WS_2 hetero-nanoribbon. **(C)** Raman spectra collected along the white line in (A) and (B), showing the spatial evolution of the MoS_2 and WS_2 characteristics peaks.

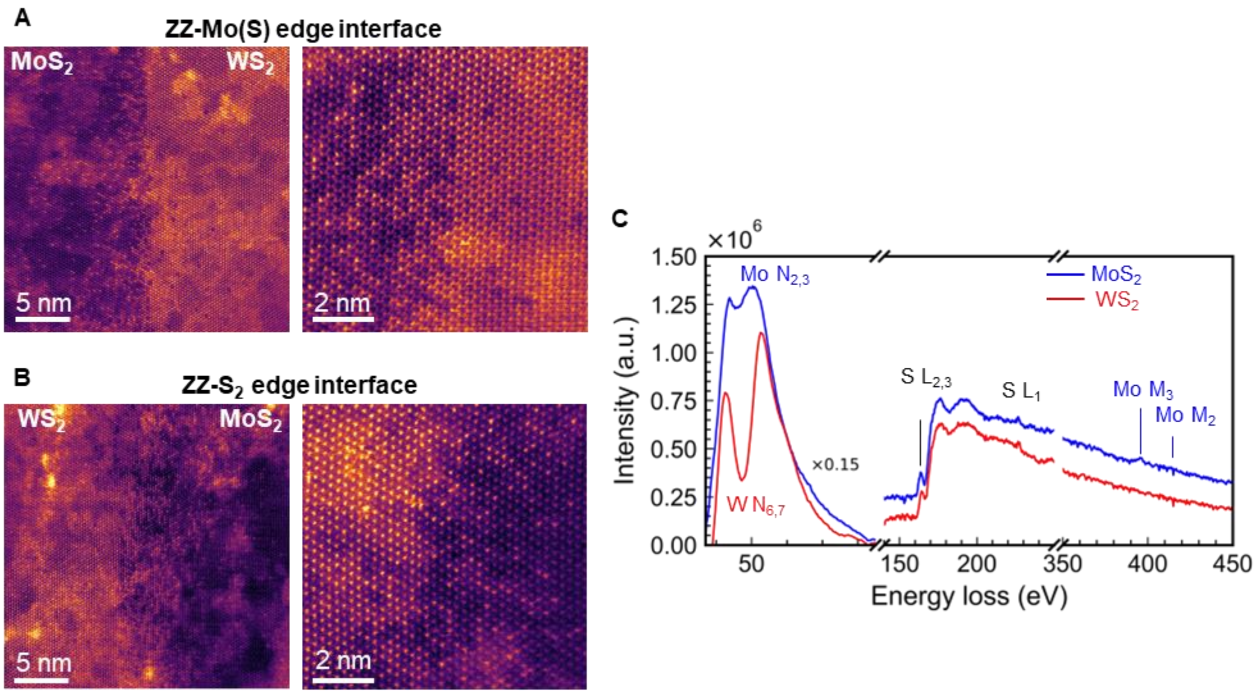

Fig. S18.
Characterization of the MoS_2-WS_2 interface of hetero-nanoribbons. (**A-B**) High resolution STEM images of the MoS_2-WS_2 interface of a hNR at the MoS_2 ZZ-Mo(S) (A) and ZZ-S_2 (B) termination edges. The interface at the smoother ZZ-Mo(S) edge of the MoS_2 has a smaller diffusion region. Brighter and darker atoms correspond to W and Mo, respectively. (**C**) EELS spectra of the MoS_2 and WS_2 regions of a MoS_2-WS_2 hNR.

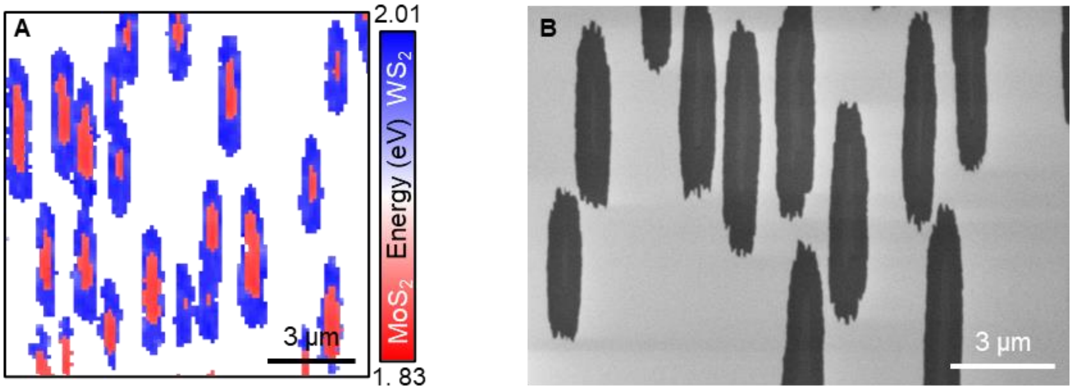

Fig. S19.
Wide-area characterization of the hetero-nanoribbons. (**A**) Wide-area PL energy mapping of as-grown MoS_2-WS_2 hNRs, corresponding to the same area as that of Fig. 4H. (**B**) SEM image of as-grown MoS_2-WS_2 hNRs, showing a contrast between the MoS_2 seed at the center, and the WS_2 surrounding it.

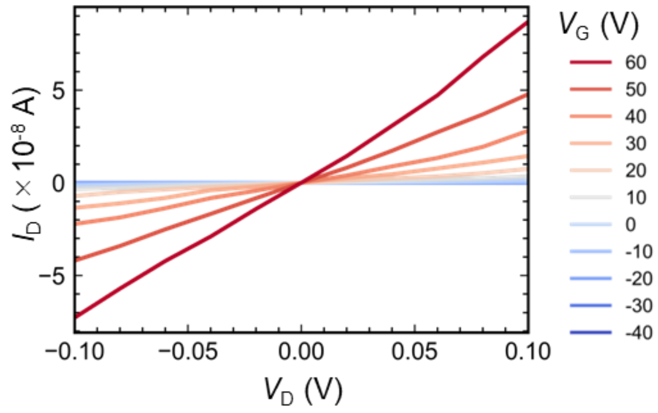

Fig. S20.

Output characteristics of the MoS_2 NR FET of Fig. 6B, measured at room temperature.

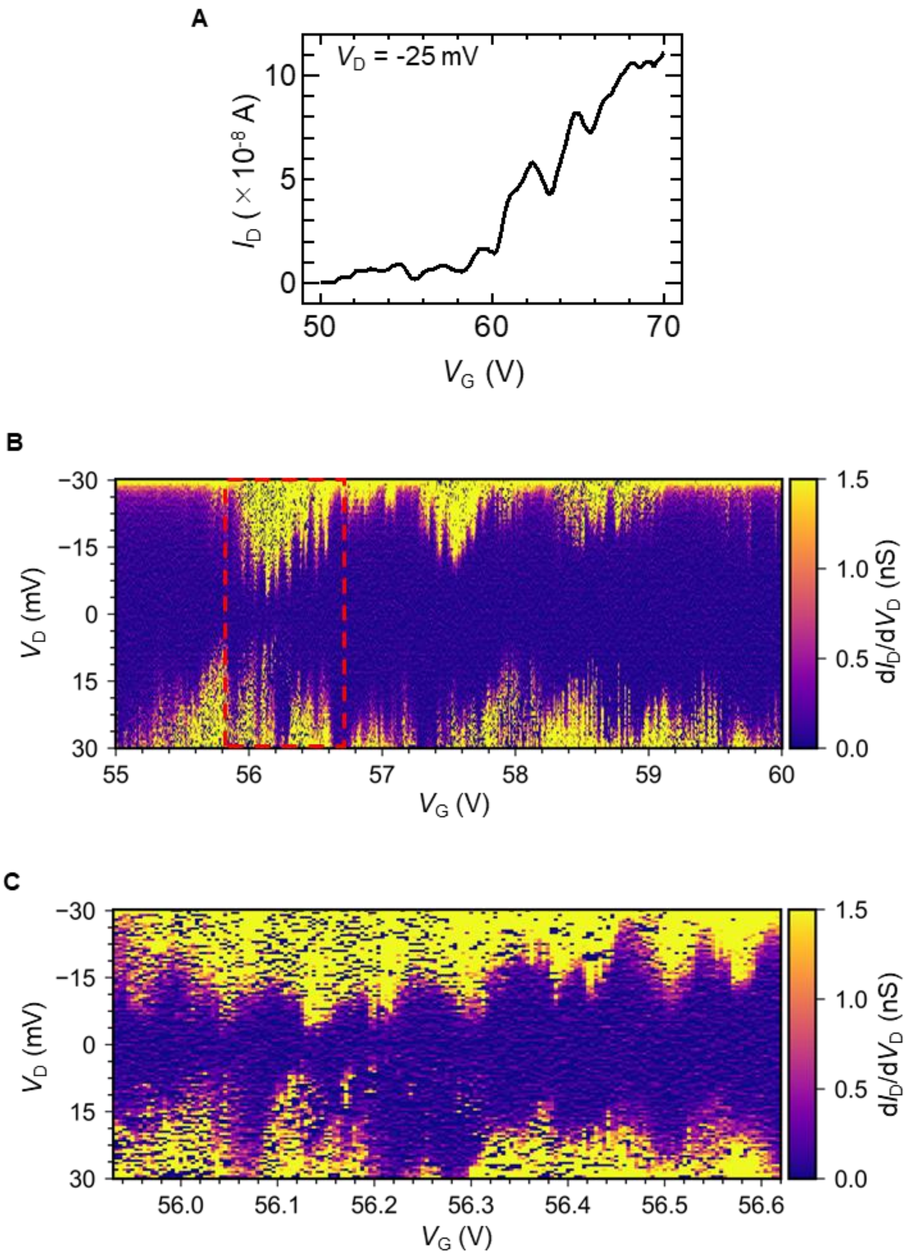

Fig. S21.

Low-temperature electrical characterization of the MoS_2 nanoribbons. (**A-B**) Typical low-temperature (4.2 K) transfer characteristics (**A**) and wide-range Coulomb diamond features (**B**) of a device with a NR channel of width 30 nm. (**C**) Enlargement of the area highlighted in (B), showing the short-period Coulomb diamond features of the device.

	Tafel slope by SECCM (mV/dec)	Tafel slope by bulk (mV/dec)
**MoS_2 NR**	139 (current results)	93 (*59*)
**MoS_2**	130 (edge) (*48*)	110 (*60*)
**$MoSe_2$**	Not found in the literature	141 (*61*)
**WS_2**	185 (terrace) (*48*)	139 (*62*)
**WSe_2**	Not found in the literature	102 (*62*)
**Pt**	105 (*63*), 40 (*64*)	30 (*60*), 30 (low overpotential) (*65*), 120 (high overpotential) (*65*)
**Pd**	Not found in the literature	30 (*65*), 121 (*66*)

Table S1.

Comparison of Tafel slope values measured by SECCM and by bulk measurement, for MoS_2 NRs, flakes of other transition metal dichalcogenides, and for Pt and Pd. The numbers in parenthesis are the corresponding references from which the values were obtained.

Movie S1.

The total energy of a trapezoidal-shaped NR-like MoS_2 flake on top of a-plane sapphire, computed across a range of relative positions of the flake over the sapphire lattice. The flake is moved along the $[1\bar{1}00]$ direction with a low energy barrier (left side) and along the $[0001]$ direction with a high energy barrier (right side).

REFERENCES AND NOTES

1. D. Akinwande, C. Huyghebaert, C.-H. Wang, M. I. Serna, S. Goossens, L.-J. Li, H.-S. P. Wong, F. H. L. Koppens, Graphene and two-dimensional materials for silicon technology. *Nature* **573**, 507–518 (2019).

2. Y. Liu, X. Duan, H.-J. Shin, S. Park, Y. Huang, X. Duan, Promises and prospects of two-dimensional transistors. *Nature* **591**, 43–53 (2021).

3. S. Wang, X. Liu, M. Xu, L. Liu, D. Yang, P. Zhou, Two-dimensional devices and integration towards the silicon lines. *Nat. Mater.* **21**, 1225–1239 (2022).

4. W. Fu, M. John, T. D. Maddumapatabandi, F. Bussolotti, Y. S. Yau, M. Lin, K. E. Johnson Goh, Toward edge engineering of two-dimensional layered transition-metal dichalcogenides by chemical vapor deposition. *ACS Nano* **17**, 16348–16368 (2023).

5. Y. Li, Z. Zhou, S. Zhang, Z. Chen, MoS_2 nanoribbons: High stability and unusual electronic and magnetic properties. *J. Am. Chem. Soc.* **130**, 16739–16744 (2008).

6. P. Cui, J.-H. Choi, W. Chen, J. Zeng, C.-K. Shih, Z. Li, Z. Zhang, Contrasting structural reconstructions, electronic properties, and magnetic orderings along different edges of zigzag transition metal dichalcogenide nanoribbons. *Nano Lett.* **17**, 1097–1101 (2017).

7. M. Maruyama, Y. Gao, A. Yamanaka, S. Okada, Geometric structure and piezoelectric polarization of MoS_2 nanoribbons under uniaxial strain. *FlatChem* **29**, 100289 (2021).

8. Y. Cai, G. Zhang, Y.-W. Zhang, Polarity-reversed robust carrier mobility in monolayer MoS_2 nanoribbons. *J. Am. Chem. Soc.* **136**, 6269–6275 (2014).

9. H. I. Karunadasa, E. Montalvo, Y. Sun, M. Majda, J. R. Long, C. J. Chang, A molecular MoS_2 edge site mimic for catalytic hydrogen generation. *Science* **335**, 698–702 (2012).

10. Z. Li, M. Huang, J. Li, H. Zhu, Large-scale, controllable synthesis of ultrathin platinum diselenide ribbons for efficient electrocatalytic hydrogen evolution. *Adv. Funct. Mater.* **33**, 2300376 (2023).

11. S. Chen, S. Kim, W. Chen, J. Yuan, R. Bashir, J. Lou, A. M. van der Zande, W. P. King, Monolayer MoS_2 nanoribbon transistors fabricated by scanning probe lithography. *Nano Lett.* **19**, 2092–2098 (2019).

12. D. Kotekar-Patil, J. Deng, S. L. Wong, C. S. Lau, K. E. J. Goh, Single layer MoS_2 nanoribbon field effect transistor. *Appl. Phys. Lett.* **114**, 013508 (2019).

13. M. A. Aslam, T. H. Tran, A. Supina, O. Siri, V. Meunier, K. Watanabe, T. Taniguchi, M. Kralj, C. Teichert, E. Sheremet, R. D. Rodriguez, A. Matković, Single-crystalline nanoribbon network field effect transistors from arbitrary two-dimensional materials. *NPJ 2D Mater. Appl.* **6**, 76 (2022).

14. R. Canton-Vitoria, T. Hotta, M. Xue, S. Zhang, R. Kitaura, Synthesis and characterization of transition metal dichalcogenide nanoribbons based on a controllable O_2 etching. *JACS Au* **3**, 775–784 (2023).

15. R. T. K. Schock, J. Neuwald, W. Möckel, M. Kronseder, L. Pirker, M. Remškar, A. K. Hüttel, Non-destructive low-temperature contacts to MoS_2 nanoribbon and nanotube quantum dots. *Adv. Mater.* **35**, 2209333 (2023).

16. S. Padmajan Sasikala, Y. Singh, L. Bing, T. Yun, S. H. Koo, Y. Jung, S. O. Kim, Longitudinal unzipping of 2D transition metal dichalcogenides. *Nat. Commun.* **11**, 5032 (2020).

17. A. P. Saunders, V. Chen, J. Wang, Q. Li, A. C. Johnson, A. S. McKeown-Green, H. J. Zeng, T. K. Mac, M. T. Trinh, T. F. Heinz, E. Pop, F. Liu, Direct exfoliation of nanoribbons from bulk van der Waals crystals. *Small* **20**, 2403504 (2024).

18. J.-H. Fu, J. Min, C.-K. Chang, C.-C. Tseng, Q. Wang, H. Sugisaki, C. Li, Y.-M. Chang, I. Alnami, W.-R. Syong, C. Lin, F. Fang, L. Zhao, T.-H. Lo, C.-S. Lai, W.-S. Chiu, Z.-S. Jian, W.-H. Chang, Y.-J. Lu, K. Shih, L.-J. Li, Y. Wan, Y. Shi, V. Tung, Oriented lateral growth of two-dimensional materials on c-plane sapphire. *Nat. Nanotechnol.* **18**, 1289–1294 (2023).

19. S. Li, Y.-C. Lin, W. Zhao, J. Wu, Z. Wang, Z. Hu, Y. Shen, D.-M. Tang, J. Wang, Q. Zhang, H. Zhu, L. Chu, W. Zhao, C. Liu, Z. Sun, T. Taniguchi, M. Osada, W. Chen, Q.-H. Xu, A. T.

S. Wee, K. Suenaga, F. Ding, G. Eda, Vapour–liquid–solid growth of monolayer MoS_2 nanoribbons. *Nat. Mater.* **17**, 535–542 (2018).

20. X. Li, B. Li, J. Lei, K. V. Bets, X. Sang, E. Okogbue, Y. Liu, R. R. Unocic, B. I. Yakobson, J. Hone, A. R. Harutyunyan, Nickel particle–enabled width-controlled growth of bilayer molybdenum disulfide nanoribbons. *Sci. Adv.* **7**, eabk1892 (2021).

21. T. Chowdhury, J. Kim, E. C. Sadler, C. Li, S. W. Lee, K. Jo, W. Xu, D. H. Gracias, N. V. Drichko, D. Jariwala, T. H. Brintlinger, T. Mueller, H.-G. Park, T. J. Kempa, Substrate-directed synthesis of MoS_2 nanocrystals with tunable dimensionality and optical properties. *Nat. Nanotechnol.* **15**, 29–34 (2020).

22. A. Aljarb, J.-H. Fu, C.-C. Hsu, C.-P. Chuu, Y. Wan, M. Hakami, D. R. Naphade, E. Yengel, C.-J. Lee, S. Brems, T.-A. Chen, M.-Y. Li, S.-H. Bae, W.-T. Hsu, Z. Cao, R. Albaridy, S. Lopatin, W.-H. Chang, T. D. Anthopoulos, J. Kim, L.-J. Li, V. Tung, Ledge-directed epitaxy of continuously self-aligned single-crystalline nanoribbons of transition metal dichalcogenides. *Nat. Mater.* **19**, 1300–1306 (2020).

23. P. Yang, D. Wang, X. Zhao, W. Quan, Q. Jiang, X. Li, B. Tang, J. Hu, L. Zhu, S. Pan, Y. Shi, Y. Huan, F. Cui, S. Qiao, Q. Chen, Z. Liu, X. Zou, Y. Zhang, Epitaxial growth of inch-scale single-crystal transition metal dichalcogenides through the patching of unidirectionally orientated ribbons. *Nat. Commun.* **13**, 3238 (2022).

24. H. Xu, S. Liu, Z. Ding, S. J. R. Tan, K. M. Yam, Y. Bao, C. T. Nai, M.-F. Ng, J. Lu, C. Zhang, K. P. Loh, Oscillating edge states in one-dimensional MoS_2 nanowires. *Nat. Commun.* **7**, 12904 (2016).

25. S. Han, X. Liu, C. Zhou, Template-free directional growth of single-walled carbon nanotubes on a- and r-plane sapphire. *J. Am. Chem. Soc.* **127**, 5294–5295 (2005).

26. H. Ago, N. Uehara, K. Ikeda, R. Ohdo, K. Nakamura, M. Tsuji, Synthesis of horizontally-aligned single-walled carbon nanotubes with controllable density on sapphire surface and polarized Raman spectroscopy. *Chem. Phys. Lett.* **421**, 399–403 (2006).

27. P. Solís-Fernández, K. Yoshida, Y. Ogawa, M. Tsuji, H. Ago, Dense arrays of highly aligned graphene nanoribbons produced by substrate-controlled metal-assisted etching of graphene. *Adv. Mater.* **25**, 6562–6568 (2013).

28. Z. Ma, S. Wang, Q. Deng, Z. Hou, X. Zhou, X. Li, F. Cui, H. Si, T. Zhai, H. Xu, Epitaxial growth of rectangle shape MoS_2 with highly aligned orientation on twofold symmetry a-plane sapphire. *Small* **16**, e2000596 (2020).

29. J. I. Sohn, H. J. Joo, A. E. Porter, C.-J. Choi, K. Kim, D. J. Kang, M. E. Welland, Direct observation of the structural component of the metal-insulator phase transition and growth habits of epitaxially grown VO_2 nanowires. *Nano Lett.* **7**, 1570–1574 (2007).

30. H. Ago, Y. Nakamura, Y. Ogawa, M. Tsuji, Combinatorial catalyst approach for high-density growth of horizontally aligned single-walled carbon nanotubes on sapphire. *Carbon* **49**, 176–186 (2011).

31. J. Wang, X. Xu, T. Cheng, L. Gu, R. Qiao, Z. Liang, D. Ding, H. Hong, P. Zheng, Z. Zhang, Z. Zhang, S. Zhang, G. Cui, C. Chang, C. Huang, J. Qi, J. Liang, C. Liu, Y. Zuo, G. Xue, X. Fang, J. Tian, M. Wu, Y. Guo, Z. Yao, Q. Jiao, L. Liu, P. Gao, Q. Li, R. Yang, G. Zhang, Z. Tang, D. Yu, E. Wang, J. Lu, Y. Zhao, S. Wu, F. Ding, K. Liu, Dual-coupling-guided epitaxial growth of wafer-scale single-crystal WS_2 monolayer on vicinal a-plane sapphire. *Nat. Nanotechnol.* **17**, 33–38 (2022).

32. C. Lee, H. Yan, L. E. Brus, T. F. Heinz, J. Hone, S. Ryu, Anomalous lattice vibrations of single- and few-layer MoS_2. *ACS Nano* **4**, 2695–2700 (2010).

33. Z. Liu, M. Amani, S. Najmaei, Q. Xu, X. Zou, W. Zhou, T. Yu, C. Qiu, A. G. Birdwell, F. J. Crowne, R. Vajtai, B. I. Yakobson, Z. Xia, M. Dubey, P. M. Ajayan, J. Lou, Strain and structure heterogeneity in MoS_2 atomic layers grown by chemical vapour deposition. *Nat. Commun.* **5**, 5246 (2014).

34. A.-Y. Lu, L. G. P. Martins, P.-C. Shen, Z. Chen, J.-H. Park, M. Xue, J. Han, N. Mao, M.-H. Chiu, T. Palacios, V. Tung, J. Kong, Unraveling the correlation between Raman and

photoluminescence in monolayer MoS_2 through machine learning models. *Adv. Mater.* **34**, e2202911 (2022).

35. Y. Li, Y. Rao, K. F. Mak, Y. You, S. Wang, C. R. Dean, T. F. Heinz, Probing symmetry properties of few-layer MoS_2 and h-BN by optical second-harmonic generation. *Nano Lett.* **13**, 3329–3333 (2013).

36. Y. Ding, X. Wang, Q. Xu, Z. Xiong, H. Wang, Y. Yu, T. Zhang, S. Zhang, M. Zeng, L. Fu, Transition metal dichalcogenides heterostructures nanoribbons. *ACS Mater. Lett.* **5**, 1781–1786 (2023).

37. J. V. Lauritsen, J. Kibsgaard, S. Helveg, H. Topsøe, B. S. Clausen, E. Lægsgaard, F. Besenbacher, Size-dependent structure of MoS_2 nanocrystals. *Nat. Nanotechnol.* **2**, 53–58 (2007).

38. X. Zhao, D. Fu, Z. Ding, Y.-Y. Zhang, D. Wan, S. J. R. Tan, Z. Chen, K. Leng, J. Dan, W. Fu, D. Geng, P. Song, Y. Du, T. Venkatesan, S. T. Pantelides, S. J. Pennycook, W. Zhou, K. P. Loh, Mo-terminated edge reconstructions in nanoporous molybdenum disulfide film. *Nano Lett.* **18**, 482–490 (2018).

39. S. Zhu, Q. Wang, A simple method for understanding the triangular growth patterns of transition metal dichalcogenide sheets. *AIP Adv.* **5**, 107105 (2015).

40. D. Cao, T. Shen, P. Liang, X. Chen, H. Shu, Role of chemical potential in flake shape and edge properties of monolayer MoS_2. *J. Phys. Chem. C* **119**, 4294–4301 (2015).

41. Y. Chen, P. Cui, X. Ren, C. Zhang, C. Jin, Z. Zhang, C.-K. Shih, Fabrication of $MoSe_2$ nanoribbons via an unusual morphological phase transition. *Nat. Commun.* **8**, 15135 (2017).

42. J. Wang, Y. Luo, X. Cai, R. Shi, W. Wang, T. Li, Z. Wu, X. Zhang, O. Peng, A. Amini, C. Tang, K. Liu, N. Wang, C. Cheng, Multiple regulation over growth direction, band structure, and dimension of monolayer WS_2 by a quartz substrate. *Chem. Mater.* **32**, 2508–2517 (2020).

43. J. Wang, Z. Li, H. Chen, G. Deng, X. Niu, Recent advances in 2D lateral heterostructures. *Nanomicro Lett.* **11**, 48 (2019).

44. Y. Gong, J. Lin, X. Wang, G. Shi, S. Lei, Z. Lin, X. Zou, G. Ye, R. Vajtai, B. I. Yakobson, H. Terrones, M. Terrones, B. K. Tay, J. Lou, S. T. Pantelides, Z. Liu, W. Zhou, P. M. Ajayan, Vertical and in-plane heterostructures from WS_2/MoS_2 monolayers. *Nat. Mater.* **13**, 1135–1142 (2014).

45. M.-Y. Li, Y. Shi, C.-C. Cheng, L.-S. Lu, Y.-C. Lin, H.-L. Tang, M.-L. Tsai, C.-W. Chu, K.-H. Wei, J.-H. He, W.-H. Chang, K. Suenaga, L.-J. Li, Epitaxial growth of a monolayer WSe_2-MoS_2 lateral p-n junction with an atomically sharp interface. *Science* **349**, 524–528 (2015).

46. Z. Huang, H. Zhao, S. Liu, X. Liang, Y. Chen, Z. Wu, X. Liu, H. Yuan, C. Wang, X. Shi, Broad band modulation of two-dimensional $Mo_{1-x}W_xS_2$ by variational compositions. *J. Phys. D Appl. Phys.* **57**, 315109 (2024).

47. J. Zhou, B. Tang, J. Lin, D. Lv, J. Shi, L. Sun, Q. Zeng, L. Niu, F. Liu, X. Wang, X. Liu, K. Suenaga, C. Jin, Z. Liu, Morphology engineering in monolayer MoS_2-WS_2 lateral heterostructures. *Adv. Funct. Mater.* **28**, 1801568 (2018).

48. Y. Takahashi, Y. Kobayashi, Z. Wang, Y. Ito, M. Ota, H. Ida, A. Kumatani, K. Miyazawa, T. Fujita, H. Shiku, Y. E. Korchev, Y. Miyata, T. Fukuma, M. Chen, T. Matsue, High-resolution electrochemical mapping of the hydrogen evolution reaction on transition-metal dichalcogenide nanosheets. *Angew. Chem. Int. Ed. Engl.* **59**, 3601–3608 (2020).

49. D. Lembke, A. Kis, Breakdown of high-performance monolayer MoS_2 transistors. *ACS Nano* **6**, 10070–10075 (2012).

50. T. Kato, T. Kitada, M. Seo, W. Okita, N. Sato, M. Shinozaki, T. Abe, T. Kumasaka, T. Aizawa, Y. Muto, T. Kaneko, T. Otsuka, Scalable fabrication of graphene nanoribbon quantum dot devices with stable orbital-level spacing. *Commun. Mater.* **3**, 103 (2022).

51. Y.-M. Lin, V. Perebeinos, Z. Chen, P. Avouris, Electrical observation of subband formation in graphene nanoribbons. *Phys. Rev. B* **78**, 161409 (2008).

52. C. M. Orofeo, H. Hibino, K. Kawahara, Y. Ogawa, M. Tsuji, K. Ikeda, S. Mizuno, H. Ago, Influence of Cu metal on the domain structure and carrier mobility in single-layer graphene. *Carbon* **50**, 2189–2196 (2012).

53. J. Barthel, Dr. Probe: A software for high-resolution STEM image simulation. *Ultramicroscopy* **193**, 1–11 (2018).

54. W. Kohn, L. J. Sham, Self-consistent equations including exchange and correlation effects. *Phys. Rev.* **140**, A1133–A1138 (1965).

55. Y. Morikawa, K. Iwata, K. Terakura, Theoretical study of hydrogenation process of formate on clean and Zn deposited Cu (1 1 1) surfaces. *Appl. Surf. Sci.* **169–170**, 11–15 (2001).

56. V. R. Cooper, Van der Waals density functional: An appropriate exchange functional. *Phys. Rev. B* **81**, 161104 (2010).

57. D. Vanderbilt, Soft self-consistent pseudopotentials in a generalized eigenvalue formalism. *Phys. Rev. B* **41**, 7892–7895 (1990).

58. M. Otani, O. Sugino, First-principles calculations of charged surfaces and interfaces: A plane-wave nonrepeated slab approach. *Phys. Rev. B* **73**, 115407 (2006).

59. D.-R. Chen, J. Muthu, X.-Y. Guo, H.-T. Chin, Y.-C. Lin, G. Haider, C.-C. Ting, M. Kalbac, M. Hofmann, Y.-P. Hsieh, Edge-dominated hydrogen evolution reactions in ultra-narrow MoS_2 nanoribbon arrays. *J. Mater. Chem. A* **11**, 15802–15810 (2023).

60. M. A. Lukowski, A. S. Daniel, F. Meng, A. Forticaux, L. Li, S. Jin, Enhanced hydrogen evolution catalysis from chemically exfoliated metallic MoS_2 nanosheets. *J. Am. Chem. Soc.* **135**, 10274–10277 (2013).

61. M. Jiang, J. Zhang, M. Wu, W. Jian, H. Xue, T.-W. Ng, C.-S. Lee, J. Xu, Synthesis of 1T-$MoSe_2$ ultrathin nanosheets with an expanded interlayer spacing of 1.17 nm for efficient hydrogen evolution reaction. *J. Mater. Chem. A* **4**, 14949–14953 (2016).

62. B. Rehman, K. M. M. D. K. Kimbulapitiya, M. Date, C.-T. Chen, R.-H. Cyu, Y.-R. Peng, M. Chaudhary, F.-C. Chuang, Y.-L. Chueh, Rational design of phase-engineered WS_2/WSe_2 heterostructures by low-temperature plasma-assisted sulfurization and selenization toward enhanced HER performance. *ACS Appl. Mater. Interfaces* **16**, 32490–32502 (2024).

63. Y. Wang, M. Li, H. Ren, Voltammetric mapping of hydrogen evolution reaction on Pt locally via scanning electrochemical cell microscopy. *ACS Meas. Sci. Au* **2**, 304–308 (2022).

64. E. B. Tetteh, M. Kim, A. Savan, A. Ludwig, T. D. Chung, W. Schuhmann, Reassessing the intrinsic hydrogen evolution reaction activity of platinum using scanning electrochemical cell microscopy. *Cell Rep. Phys. Sci.* **4**, 101680 (2023).

65. S. Sarkar, S. C. Peter, An overview on Pd-based electrocatalysts for the hydrogen evolution reaction. *Inorg. Chem. Front.* **5**, 2060–2080 (2018).

66. T. Chao, X. Luo, W. Chen, B. Jiang, J. Ge, Y. Lin, G. Wu, X. Wang, Y. Hu, Z. Zhuang, Y. Wu, X. Hong, Y. Li, Atomically dispersed copper–platinum dual sites alloyed with palladium nanorings catalyze the hydrogen evolution reaction. *Angew. Chem. Int. Ed. Engl.* **56**, 16047–16051 (2017).
